# Enhanced Zn/ZnO Heterointerfaces via Pulsed-Potential Electrochemical Reconstruction for Highly Selective CO_2_ Reduction

**DOI:** 10.1021/acsami.5c11775

**Published:** 2025-10-02

**Authors:** Hsin-Chiao Wu, Yu-Wei Huang, Yu-Chang Lin, Chu-Hsin Yang, Ta-Chung Liu

**Affiliations:** † Department of Biomedical Engineering, 34914 National Yang Ming Chiao Tung University, Taipei 112, Taiwan; ‡ 57815 National Synchrotron Radiation Research Center, Hsinchu 30076, Taiwan

**Keywords:** heterointerface, pulsed potential, CO_2_ reduction, Zn/ZnO, reconstruction

## Abstract

The electrochemical carbon dioxide reduction reaction (CO_2_RR) demands advanced low-cost cathodes that can overcome the intrinsic scaling limits of single-phase metals. Accordingly, we report a pulsed-potential-based square wave voltammetry (SWV) strategy to reconstruct ZnO nanoparticles into metallic Zn nanoislands embedded on the ZnO wurtzite surface, generating an enhanced density of Zn/ZnO heterointerfaces. TEM/FFT and XPS analyses confirmed more finely discrete metallic Zn domains with a balanced Zn(0)/Zn­(II) ratio for the SWV-ZnO electrocatalyst. At these heterojunctions, they preferentially stabilized the *COOH intermediate and weakened *H adsorption, thereby suppressing the competing hydrogen evolution reaction. The SWV-ZnO delivered a peak CO Faradaic efficiency of 90% with a CO partial current density of 5.3 mA/cm^2^ at −1.05 V versus RHE in the H-cell, outperforming the pristine zinc nanoparticles and the previously reported potentiostatic reconstruction strategies (CA-ZnO). This work demonstrated that pulsed-potential electrochemical reconstruction offered a rapid and scalable route to engineer heterointerfaces, providing a practical blueprint for advancing sustainable CO_2_-to-CO conversion technologies.

## Graphical abstract

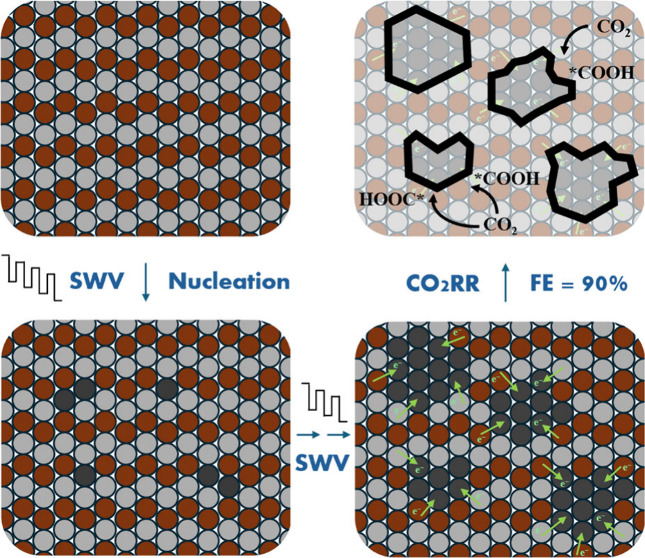

## 1 Introduction

As anthropogenic carbon dioxide emissions continue to increase, the development of environmentally benign mitigation technologies has become urgent.^1,2^ The electrochemical reduction of carbon dioxide (CO_2_RR) has gained prominence because it converts CO_2_ into value-added chemicals at ambient temperature and pressure.^3^ The catalyst selected for the cathode governs both the nature and proportion of the products by virtue of its composition and surface properties.^4^ In aqueous media, certain noble metals possess nearly ideal adsorption free energies for key CO_2_RR intermediates, as predicted by the Sabatier principle, and consequently favor the CO_2_RR over the competing hydrogen evolution reaction (HER).^5,6^ Typical examples include gold and silver, which selectively yield carbon monoxide,^7,8^ and palladium, which produces formic acid at comparatively low overpotentials.^9^

Single-phase catalysts for the CO_2_RR are fundamentally constrained by linear scaling relations, in which the adsorption of free energies of chemically related intermediates (e.g., *COOH and *CO) varies in lock step with a single surface descriptor.^10,11^ Because strengthening the binding of one intermediate automatically strengthens (or weakens) the other, no single metal can simultaneously minimize all thermodynamic barriers, giving rise to the classic volcano limitation.^12^ However, heterointerfaces provide a direct route to break these constraints. For example, Tang et al. anchored Ag nanoparticles on CeO_2_ nanorods using an ultrasonic loading method. Oxygen vacancies triggered directional electron spillover at the Ag/O_V_-CeO_2_ interface, generating negatively charged Ce^3+^-O_V_ domains alongside mildly electron-depleted Ag sites.^13^ The *COOH intermediate thus gained simultaneous σ coordination and electrostatic stabilization at these Ag-Ce^3+^ dual sites. Similarly, Qi et al. employed atomic layer deposition to coat TiO_2_ onto MoS_2_, forming TiO_2_/MoS_2_ heterointerfaces.^14^ The TiO_2_/MoS_2_ heterointerface furnished Mo–Ti dual active sites, in which Mo sites bound *CO strongly, whereas neighboring Ti sites bound it only weakly. This asymmetric coordination enabled *CO to migrate from the Mo to Ti sites and facilitated the CO–CO coupling at the Ti sites.

Electrochemical reconstruction is an exceptionally simple tool for sculpting heterointerfaces.^15,16^ During an electrochemical reconstruction step, the precursor oxide is held at a reducing potential, allowing metal nucleation to generate metal/oxide heterointerfaces without elaborate vacuum or thermal steps. For example, Ning et al. in situ reduced the bottom layers of SnO_2_ to metallic Sn by applying a constant potential of −1.0 V versus RHE.^17^ The resulting Sn/SnO_2_ Mott–Schottky interface and the built-in electric field selectively stabilized the *HCOO intermediate while suppressing the HER. Consequently, the FE for formate increased from 44% to 93.7%. Yan et al. used similar strategies to reduce a CuO precursor preloaded on CeO_2_ into ultrafine Cu_2_O nanodomains that formed a coherent Cu_2_O/Ce_2_O heterointerface.^18^ CeO_2_ promoted the simultaneous adsorption of CO_2_ and protons, thereby accelerating their electrocatalytic conversion from CO_2_ to CH_4_ at neighboring Cu^+^ active sites.

A massive amount of research has expanded the portfolio of CO_2_RR catalysts well beyond noble metals, and a number of Earth-abundant transition metals show promising activity.^19,20^ Among these, zinc and its derived metallic zinc compound phases have emerged as a leading candidate for the selective CO_2_RR to CO, positioning low-cost but effective catalyst design for CO_2_RR systems at the forefront.^21,22^ Accordingly, constructing Zn/ZnO heterointerfaces became our central objective. However, conventional chronoamperometric (CA) reconstruction at highly negative potentials can overreduce ZnO, which makes alternative electrochemical reconstruction strategies imperative.

In this work, we report a pulsed-potential-based electrochemical reconstruction route to generate rich Zn/ZnO heterointerfaces. The rapid potential pulses inherent to square wave voltammetry (SWV) induced the nucleation of scattered Zn nanoislands on the ZnO surface. XPS and TEM/FFT analyses revealed that SWV-treated ZnO contained uniformly dispersed Zn nanoislands in a more balanced and nonexcessive quantity compared to its CA-treated counterpart, markedly increasing the number of Zn/ZnO heterointerfaces. These heterointerfaces likely redistributed electrons in their vicinity, stabilizing key CO_2_RR intermediates such as *COOH. This structural advantage translated into superior electrocatalytic behavior. SWV-ZnO reached a CO Faradaic efficiency of 90% at −1.05 V versus RHE in the H-cell and sustained stable electrocatalytic performance for 8 h in a flow cell (FE_CO_ > 80%). These results demonstrated that pulsed-potential electrochemical reconstruction provided a facile and effective strategy to engineer high-density heterointerfaces and thereby potentially fabricate low-cost electrocatalysts with enhanced CO_2_RR performance.

## 2 Experimental Section

### 2.1 Materials and Reagents

Commercial zinc oxide nanoparticles (nanoZnO) and potassium hydroxide (KOH) were purchased from Alfa Aesar. Potassium chloride (KCl) was acquired from Uni-Bio Science Group; 36.5–38% hydrogen chloride (HCl) was bought from J. T. Baker. Dihydrogen hexachloroplatinate (H_2_PtCl_6_·*x*H_2_O) was purchased from UniRegion Bio-Tech, and 99.8% isopropyl alcohol (IPA) was obtained from Emperor Chemical Co., Ltd. The Nafion 115 membrane was supplied by MorrChem Technologies. The gas diffusion electrode (GDL-AvCarb GDS5130, GDE) was purchased from Dioxide Materials. The platinum sheet (Pt) and titanium (Ti) foam were obtained from IMGJING. Carbon dioxide (CO_2_), helium (He), and nitrogen (N_2_) were purchased from C. C. Gaseous Corp.

### 2.2 Cathode Preparation

We used an ultrasonic spray coating system to deposit 1 mg/cm^2^ of commercial ZnO nanoparticles (P-ZnO) on a 2 cm × 2 cm GDE. A peristaltic pump delivered 1 wt % P-ZnO precursor dispersed in IPA to the ultrasonic spray system at a rate of 1 mL/min. Then, the precursor was atomized onto the GDE heated at 150 °C by a hot plate with an ultrasonic power of 23 V and 0.8 A until 1 mg/cm^2^ of P-ZnO was coated. After spraying, the coated GDE cathode was placed in an oven at 50 °C for 8 h to remove residual solvents.

To obtain the SWV-ZnO electrodes, a P-ZnO cathode with a loading of 1 mg/cm^2^ was installed in the H-type cell described in section 2.3, followed by a square wave voltammetry (SWV) step. P-ZnO was converted into SWV-ZnO by cycling between −0.1 and −2.4 V versus Ag/AgCl at a frequency of 50 Hz with a pulse amplitude of 10 mV. A schematic of the SWV reconstruction procedures is shown in Figure S1. For comparison, P-ZnO was electrochemically reconstructed via chronoamperometry (CA) by holding the potential at −2.4 V versus Ag/AgCl for 20 min. The sample is denoted as CA-ZnO. Both SWV-ZnO and CA-ZnO were obtained from P-ZnO via electrochemical reconstruction for 20 min.

### 2.3 Electrochemical Measurements

Unless otherwise stated, all electrochemical measurements were conducted in the H-cell. A Nafion 115 membrane was used to separate the cathode and the anode. A 5 cm^2^ platinum foil served as the anode, and a 0.1 M KCl solution was used as the electrolyte. A Ag/AgCl electrode (BASMF2056, Sigma-Aldrich) filled with 3 M KCl was used as a reference electrode. The catholyte was purged with pure CO_2_ or N_2_ at a rate of 50 mL/min for 1 h before the cathode reconstruction step, linear-sweep voltammetry (LSV), cyclic voltammetry, and chronoamperometry were performed. The standard electrode potential (RHE) was calibrated as shown in eq 1:

1 E(RHE)=E(Ag/AgCl)+0.198+0.0591×pH

The current interruption method was used to compensate for the ohmic drop correction by determining the catholyte resistance. Electrochemical tests were carried out with an OctoStat200 R31660 potentiostat (IVIUM Technologies) controlled by Iviumsoft software. The pH value was measured with a model HI2002-01 pH meter (HANNA Instruments).

For the ECSA calculation, the double-layer capacitance (*C*_dl_) was calculated as in eq 2:

2 $$C_{\mathrm{dl}}=\frac{\mathrm{CV area}}{2\times \mathrm{scan range}\times \mathrm{scan rate}}$$

Since ECSA is proportional to *C*_dl_, the ratio of ECSA for P-ZnO, CA-ZnO, and SWV-ZnO can be rationally normalized by the ratio of their *C*_dl_ values without knowing the actual value of ECSA in square centimeters (*C*_S_).

### 2.4 Flow Cell Assembly

In this study, the durability test was conducted in a membrane electrode assembly (MEA) flow cell (Dioxide Materials, research grade 5 cm^2^ hardware for carbon dioxide electrolysis). We used a Sustainion membrane to separate the cathode and anode compartments. CO_2_ was first humidified through a humidification bottle and then delivered to the cathode at a flow rate of 30 mL/min using an EL-FLOW base gas flow meter (Bronkhorst Co.). Meanwhile, a 0.5 M solution of KOH was introduced into the anode at a rate of 3 mL/min using a PF-102 peristaltic pump (Tohama Co., Ltd.). The anode consisted of Ti foam loaded with 1 mg/cm^2^ of Pt. To prepare the Pt/Ti foam electrode, a Ti foam (3 cm × 3 cm) was immersed in 6 M HCl at 80 °C under continuous stirring for 2 h, thoroughly rinsed with acetone, and dried in an oven. Next, 10 mL of a 10% HCl solution (with isopropanol as the solvent) was mixed with 30 mg of H_2_PtCl_6_·*x*H_2_O until fully dissolved. A 100 μL aliquot of this solution was then applied to the Ti foam, which was subsequently placed in a tubular furnace (D-80, Dogger Scientific Co., Ltd.) and calcined at 500 °C for 10 min. This dipping and calcination process was repeated until the Pt loading on the Ti foam reached 1  mg/cm^2^.

### 2.5 Product Evaluation

The gaseous products from the cathode compartment were analyzed using an HP Agilent 6890 Plus GC instrument (G1530A). The gas chromatograph was equipped with a ShinCarbon ST, 100/120 mesh, 2m, 1/16 in. outside diameter, 1.0 mm inside diameter (catalog no. 19808) column operated with a thermal conductivity detector and a flame ionization detector. All liquid products were analyzed by a JEOL NMR500 instrument (ECZ500R/S1), and their yields were negligible. Gaseous products were injected into the gas chromatograph by extracting 1 mL of gas from the cathode compartment after sealed reaction for 20 min. Helium was employed as a carrier gas for CO analysis, while nitrogen served as a carrier gas for H_2_ detection. The partial current density (*j*_P_) and Faraday efficiency (FE) were obtained from eq 3:

3 $$\mathrm{FE}=j_{p}/j=\frac{tRT}{FzPVX}/j$$

where *t* is the reaction time, *R* is the ideal gas constant, *T* is the cathodic temperature, *F* is Faraday’s constant, *z* is the number of electrons transferred in the CO_2_RR equation, *P* is the cathodic pressure, *V* is the cathodic volume, *X* is the product volume fraction, and *j* is the total current density.

### 2.6 Electrode Surface pH Simulation

The surface pH of the cathode was calculated by a reaction–diffusion model developed by Gupta et al.^23^ When the CO_2_RR was conducted, the surface species could be balanced by eqs 4 and 5:

$${\mathrm{CO}}_{2\left(\mathrm{aq}\right)}+{\mathrm{OH}}^{-}\overset{\begin{array}{c} k_{1f} \end{array}}{\underset{\begin{array}{c} k_{1r} \end{array}}{\begin{array}{c} ↔ \end{array}}}{{\mathrm{HCO}}_{3}}^{-}$$ 4

5 $${{\mathrm{HCO}}_{3}}^{-}+{\mathrm{OH}}^{-}\begin{array}{c} k_{2f}\\ ↔\\ k_{2r} \end{array}{{\mathrm{CO}}_{3}}^{2-}+H_{2}O$$

where *k*_1f_ and *k*_2f_ are forward reaction rate constants and *k*_1r_ and *k*_2r_ are reverse reaction rate constants for reactions 1 and 2, respectively. We used the rate constants reported by Gupta et al.: *k*_1f_ = 5.93 × 10^3^ M^–1^ s^–1^, *k*_1r_ = 1.34 × 10^–4^ s^–1^, *k*_2f_ = 10^8^ M^–1^ s^–1^, and *k*_2r_ = 2.15 × 10^4^ M^–1^ s^–1^. In our model, we considered the diffusion layer to be 50 μm, similar to that in of Luo et al. in the H-cell,^24^ as described by eqs 6–9:

6 $$\frac{\partial \left[{\mathrm{CO}}_{2\left(\mathrm{aq}\right)}\right]}{\partial t}=D_{{\mathrm{CO}}_{2}}\frac{\partial^{2}\left[{\mathrm{CO}}_{2\left(\mathrm{aq}\right)}\right]}{\partial x^{2}}-\left[{\mathrm{CO}}_{2\left(\mathrm{aq}\right)}\right]\left[{\mathrm{OH}}^{-}\right]k_{1f}+\left[{{\mathrm{HCO}}_{3}}^{-}\right]k_{1r}$$

7 $$\frac{\partial \left[{{\mathrm{HCO}}_{3}}^{-}\right]}{\partial t}=D_{{{\mathrm{HCO}}_{3}}^{-}}\frac{\partial^{2}\left[{{\mathrm{HCO}}_{3}}^{-}\right]}{\partial x^{2}}+\left[{\mathrm{CO}}_{2\left(\mathrm{aq}\right)}\right]\left[{\mathrm{OH}}^{-}\right]k_{1f}-\left[{{\mathrm{HCO}}_{3}}^{-}\right]k_{1r}-\left[{{\mathrm{HCO}}_{3}}^{-}\right]\left[{\mathrm{OH}}^{-}\right]k_{2f}+\left[{{\mathrm{CO}}_{3}}^{2-}\right]k_{2r}$$

8 $$\frac{\partial \left[{{\mathrm{CO}}_{3}}^{2-}\right]}{\partial t}=D_{{{\mathrm{CO}}_{3}}^{2-}}\frac{\partial^{2}\left[{{\mathrm{CO}}_{3}}^{2-}\right]}{\partial x^{2}}+\left[{{\mathrm{HCO}}_{3}}^{-}\right]\left[{\mathrm{OH}}^{-}\right]k_{2f}-\left[{{\mathrm{CO}}_{3}}^{2-}\right]k_{2r}$$

9 $$\frac{\partial \left[{\mathrm{OH}}^{-}\right]}{\partial t}=D_{{OH}^{-}}\frac{\partial^{2}\left[{\mathrm{OH}}^{-}\right]}{\partial x^{2}}-\left[{\mathrm{CO}}_{2\left(\mathrm{aq}\right)}\right]\left[{\mathrm{OH}}^{-}\right]k_{1f}+\left[{{\mathrm{HCO}}_{3}}^{-}\right]k_{1r}-\left[{{\mathrm{HCO}}_{3}}^{-}\right]\left[{\mathrm{OH}}^{-}\right]k_{2f}+\left[{{\mathrm{CO}}_{3}}^{2-}\right]k_{2r}$$

where *t* is the time, *D* is the diffusion coefficient for a given species, and *x* is the position within the boundary layer. Here, *x* = 0 represented the bulk solution, and *x* = 50 μm represented the electrode surface. The CO_2_RR resulted in the CO_2consumption_ and OH^–^_formation_ as described by eqs 10 and 11:

10 $${CO}_{2\mathrm{consumption}}=\frac{j\times \mathrm{FE}}{zF}$$

11 $${{\mathrm{OH}}^{-}}_{\mathrm{formation}}=\frac{j}{F}$$

Using the boundary conditions and initial conditions suggested by Gupta et al.,^24^ the partial differential equations (eqs 6–9) could be solved to obtain the surface pH values.

### 2.7 Characterization of the Materials

The morphologies and microstructures of samples were observed by using a JEOL JSM-7600F field emission scanning electron microscope (SEM) and a JEM-2100F transmission electron microscope (TEM). A Seiko SMI3050SE dual-beam FIB was used to prepare the samples of CA-ZnO and SWV-ZnO for TEM observation. XRD/GIXRD investigations were performed with a Bruker D8 Discover X-ray diffraction system by using a CuKα beam; all samples were coated in electrode form on GDE. Zinc K-edge X-ray absorption spectroscopy (XAS) measurements were carried out at beamline 44A of the Taiwan Photon Source (TPS), located at the National Synchrotron Radiation Research Center (NSRRC) in Hsinchu. Data were collected in transmission mode by using quick-scanning X-ray absorption spectroscopy (XAS), allowing simultaneous acquisition of both X-ray absorption near-edge structure (XANES) and extended X-ray absorption fine structure (EXAFS) regions. To ensure accurate energy calibration, metallic Zn foil was used as a reference standard. The acquired EXAFS data were processed with the Athena software package, with the spectra weighted by *k* ^3^ to enhance oscillation features in *k* space. Subsequent Fourier transform analysis was applied to convert the data into R space, facilitating the investigation of bond length variations across different sample conditions. XPS spectra were recorded with a Thermo Scientific Theta Probe X-ray photoelectron spectroscope; all samples were coated in electrode form on GDE. Electron paramagnetic resonance (EPR) spectra were recorded using a Bruker EPR- plus 2017 instrument.

## 3 Results and Discussion

### 3.1 Microstructure and Near-Surface Characterization

SEM images are presented in Figure 1a–c. Figure 1a shows that P-ZnO formed a compact layer of uniform nanoparticles. In contrast, CA-ZnO and SWV-ZnO (Figure 1b,c) developed porous and branched frameworks. A closer inspection revealed that CA-ZnO carried densely packed protrusions on the pore walls (Figure S2a), whereas the branches of SWV-ZnO appeared to be relatively smooth (Figure S2b). XRD was employed to probe the crystalline structure, as displayed in Figure 1d. All three samples exhibited diffraction peaks at 31.79°, 34.41°, and 36.25°, which corresponded to the (100), (002), and (101) planes, respectively, of wurtzite ZnO.^25^ These ZnO characteristic peak intensities decreased noticeably after electrochemical reconstruction.

**Figure 1:**
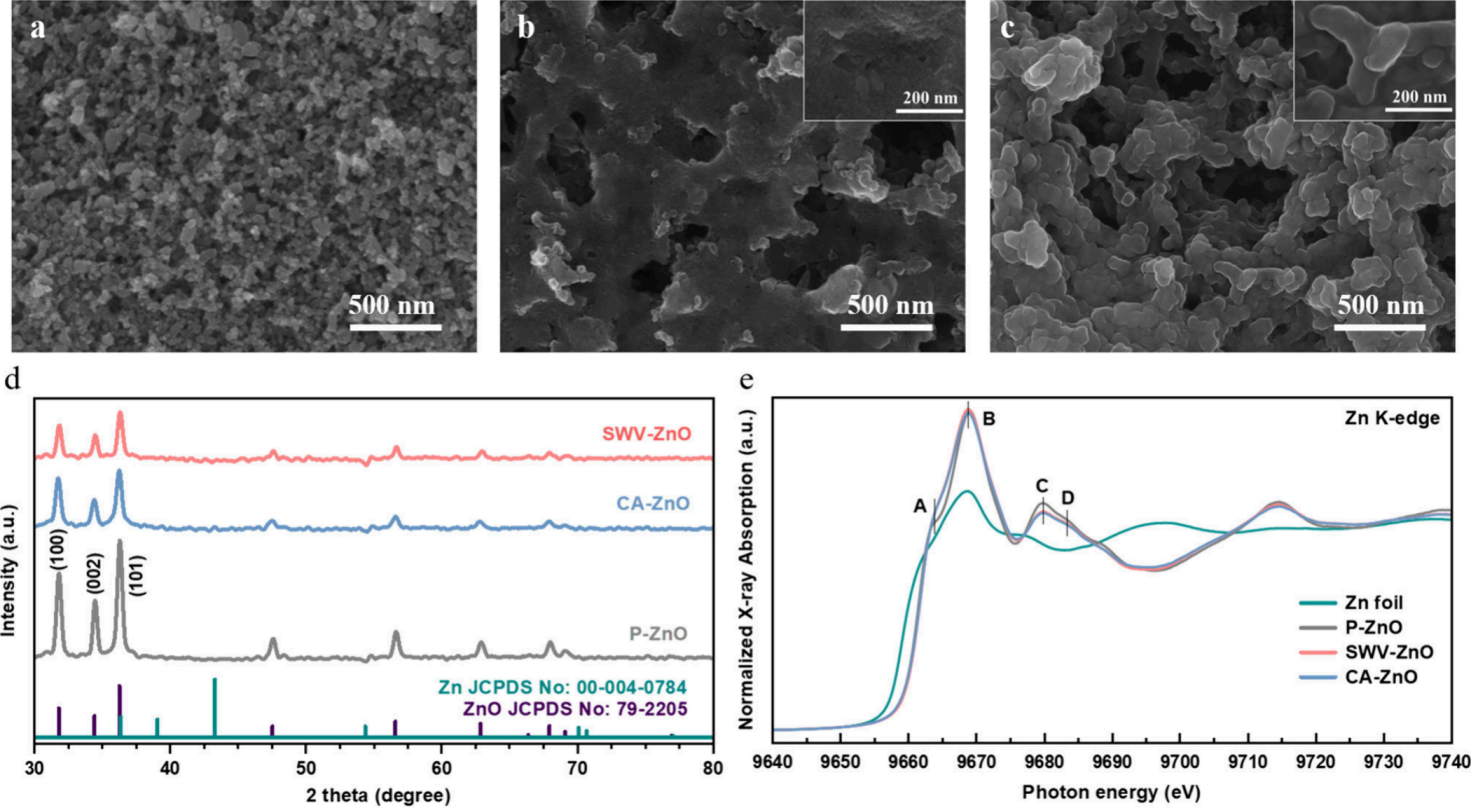
SEM images of (a) P-ZnO, (b) CA-ZnO, and (c) SWV-ZnO. (d) XRD patterns of P-ZnO, CA-ZnO, and SWV-ZnO. (e) Normalized Zn K-edge XAS spectra of the Zn foil, P-ZnO, CA-ZnO, and SWV-ZnO.

XAS was employed to determine the oxidation state and coordination of Zn in P-ZnO, CA-ZnO, and SWV-ZnO; a Zn foil spectrum served as the metallic reference (Figure 1e). A positive shift of the absorption edge is well-known to indicate a higher oxidation state.^26^ The higher-energy absorption edge observed for the three ZnO samples relative to the Zn foil, therefore, confirmed their bulk ZnO character. P-ZnO displayed a shoulder (feature A) at 9663.4 eV and a main peak (feature B) at 9668.9 eV.^27^ These were assigned to the 1s → 4sp–O 2p and 1s → 4p–O 2p transitions, respectively.^28^ A typical ZnO K-edge spectrum also contains features C and D at 9679.8 and 9683.3 eV,^27^ respectively, arising from multiple-scattering paths involving range orders of approximately 11 and 5 shells.^28^ These results confirmed that bulk material properties of CA-ZnO and SWV-ZnO were the same as those of P-ZnO.

The *k* ^3^-weighted EXAFS profiles (Figure S3a) of P-ZnO, CA-ZnO, and SWV-ZnO all display the characteristic oscillations of wurtzite ZnO, confirming that the interior lattice remains intact after electrochemical treatment. However, the *k* ^3^χ­(*k*) amplitudes for CA-ZnO and SWV-ZnO are uniformly damped throughout the *k* range of 2–12 Å^–1^, signaling a lower effective shell range order around Zn centers. Fourier-transformed EXAFS spectra (Figure S3b) resolve a Zn–O peak at ≈1.6 Å and a Zn–Zn peak at ≈2.9 Å (without phase correction). Both peaks shrink in CA-ZnO and SWV-ZnO samples compared to P-ZnO. Such attenuation may be attributed to the truncation of the shell range order by partial reduction or defect formation rather than to bulk characterization.

The TEM was further used to examine the near-surface microstructures. FIB-SEM sections of CA-ZnO and SWV-ZnO are shown in panels a and b, respectively, of Figure S4. An approximately 800 nm carbon coating formed a uniform protective layer over the porous gas diffusion electrode, beneath which lay the CA-ZnO and SWV-ZnO layers that underwent electrochemical reconstruction and the CO_2_RR. SAED patterns were collected within 50 nm of the cathode layer. Diffraction rings were indexed to the specific crystal facet of either Zn or ZnO, as marked in panel c or d, respectively, of Figure S4. For P-ZnO, the HRTEM image and its corresponding SAED pattern are provided in panels e and f, respectively, of Figure S4. In addition to the rings assigned to ZnO, both CA-ZnO and SWV-ZnO exhibited a ring that matches the (101) facet of metallic Zn, confirming the coexistence of Zn and ZnO domains and further implying the presence of Zn/ZnO heterointerfaces.^29^ More HRTEM images illustrate this phenomenon (Figure S5a,b), consistent with the SAED results. To visualize these differences, we examined HRTEM images and their FFT patterns for CA-ZnO and SWV-ZnO (Figure 2a-i,a-ii,b-i,b-ii). Both FFTs displayed the characteristic Zn(101) reflection. Points of strongest Zn(101) intensity were identified in the FFT, marked in red, blue, yellow, and green (Figures 2a-iv,b-iv), and then mapped back onto the real-space images, where they are highlighted in panels a-iii and b-iii of Figure 2. The detailed procedure for Zn(101) domain selection is provided in Note S1. Metallic Zn occupied broad contiguous patches on the CA-ZnO surface, whereas SWV-ZnO presented numerous isolated nanoscale islands. Additional HRTEM images are provided to confirm the resulting difference between CA-ZnO and SWV-ZnO (Figures S6–S11). For quantifying the size difference of new generated metallic Zn domains between the two samples, we analyzed 15 Zn(101) domains from each of the CA-ZnO and SWV-ZnO samples, measuring their long-axis dimensions and compiling the results into size distribution histograms and box plots (Figure 2c,d). The average Zn(101) domain size of SWV-ZnO was 6.20 ± 1.67 nm, whereas CA-ZnO exhibited a significantly larger average size of 11.19 ± 3.90 nm. The domain size difference via reconstruction strategies between the two samples is hypothetically attributed to the following. Under potentiostatic (CA) reconstruction, an initial burst of nucleation reduced high-energy ZnO sites to Zn, followed by lateral growth and coalescence into extended metallic regions. Pulsed-potential (SWV) reconstruction, in contrast, supplied repetitive pulses that generate a high density of discrete Zn nuclei, yielding finer metallic islands.^30^

**Figure 2:**
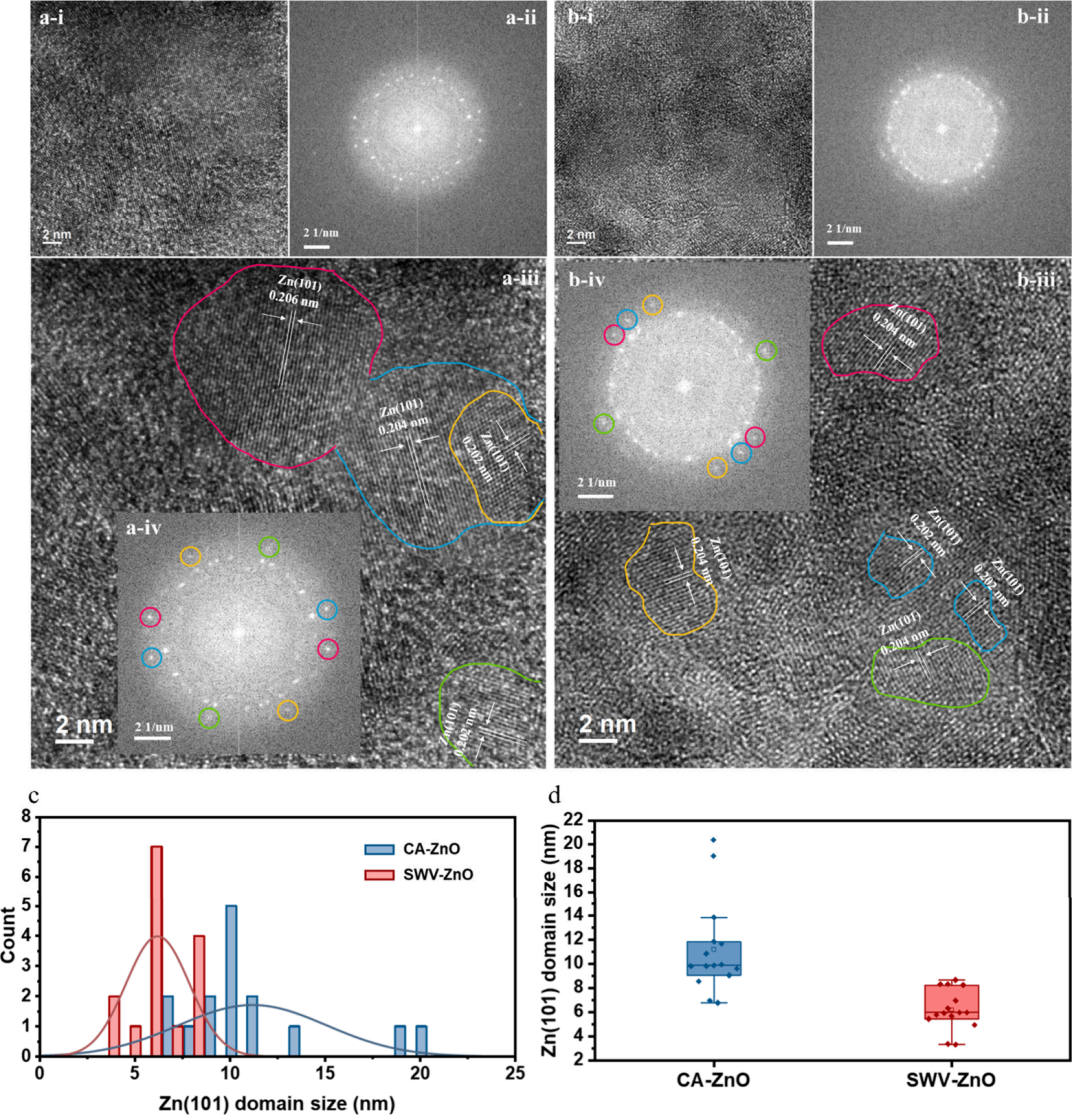
(a-i) HRTEM images, (a-ii) FFT images, and (a-iii) corresponding Zn(101) regions in HRTEM and (a-iv) FFT images for CA-ZnO. (b-i) HRTEM images, (b-ii) FFT images, and (b-iii) corresponding Zn(101) regions in HRTEM and (b-iv) FFT images for SWV-ZnO. (c) Size histograms and distributions of CA-ZnO and SWV-ZnO domains obtained from HRTEM images with a Mann–Whitney test yielding a *p* value of 0.000215. (d) Corresponding box plots.

It is hypothesized that finely discrete Zn islands within a ZnO surface can generate more Zn/ZnO heterointerfaces.^31^ The extent of reduction governs the heterointerface abundance. To determine the reduction levels on CA-ZnO and SWV-ZnO, we quantified the relative amounts of Zn(0) and Zn­(II) by deconvoluting the Zn 2p^3^ peak in the XPS spectra. The component at 1021.9 eV was assigned to Zn(0), whereas the signal at 1020.9 eV corresponded to Zn­(II).^41,42^ The fitting results for P-ZnO, CA-ZnO, and SWV-ZnO appear in Figure S12 and panels a and b of Figure 3, respectively. Ninety-three percent of the surface Zn in P-ZnO presented as Zn­(II), which confirmed that its surface was composed of well-crystallized ZnO, consistent with the ordered lattice observed by the TEM and SAED in panels e and f, respectively, of Figure S4. A clear difference emerged between the electrochemically activated samples. About 65% of the surface Zn in CA-ZnO was metallic, whereas SWV-ZnO contained only 51% Zn(0). Full XPS spectra are presented in Figure S13. These results suggested that the CA approach drove a more extensive reduction, creating larger metallic Zn regions that diminished the number of Zn/ZnO heterointerfaces. Combining the Zn 2p^3^ analysis with the HRTEM observations, SWV-ZnO attained a more moderate degree of reduction, which likely enhanced the density of the Zn/ZnO heterointerfaces.

**Figure 3:**
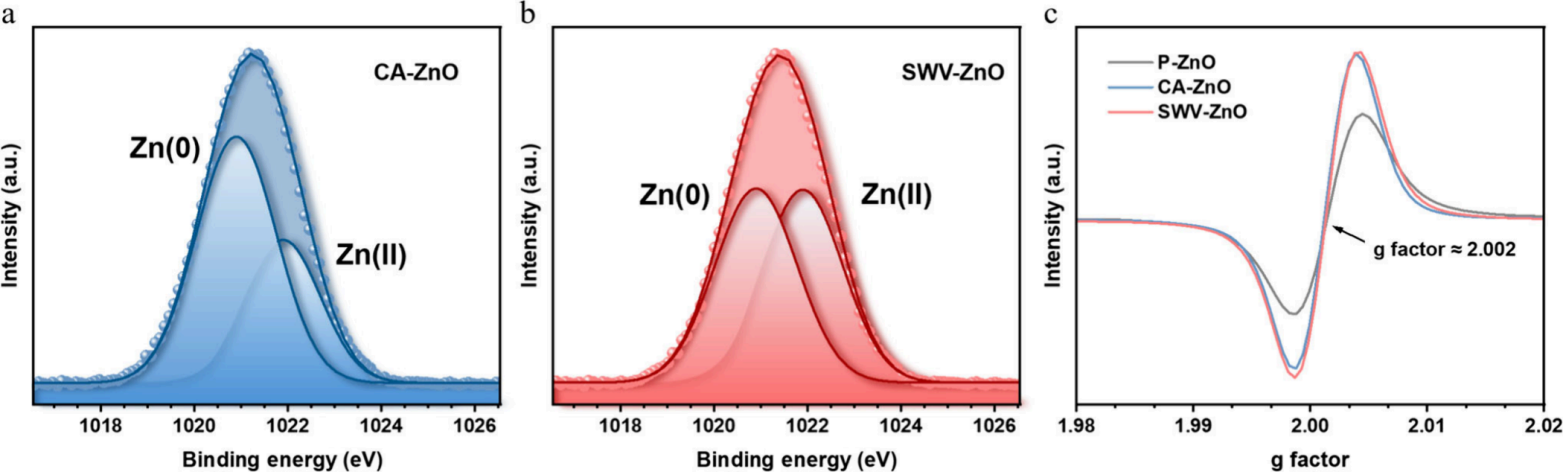
Deconvoluted Zn 2p^3^ spectra of (a) CA-ZnO and (b) SWV-ZnO, fitted with Zn(0) (1020.9 eV) and Zn­(II) (1021.9 eV). (c) EPR spectra of P-ZnO, CA-ZnO, and SWV-ZnO.

In addition, heterointerface formation is frequently accompanied by oxygen vacancies generated by the strain effect.^35^ EPR and XPS were used to investigate the oxygen defect concentrations of the samples. To probe the oxygen defect concentrations, we employed both EPR and XPS analyses. The EPR spectra are shown in Figure 3c. All samples exhibited a characteristic signal at *g* = 2.002, indicative of oxygen vacancies, where the signal intensity reflects the relative concentration of such defects.^43^ Based on Figure 3c, SWV-ZnO and CA-ZnO displayed comparable levels of oxygen vacancies, both markedly higher than those of P-ZnO. Panels a–c of Figure S14 depict the deconvoluted O 1s spectra of P-ZnO, CA-ZnO, and SWV-ZnO, respectively. Peaks at 530.2, 531.4, and 532.6 eV were assigned to lattice oxygen (O_L_), vacancy oxygen (O_V_), and adsorbed oxygen (O_C_), respectively, and their contributions were quantified.^36^ P-ZnO contained the highest proportion of O_L_ (60%), whereas CA-ZnO and SWV-ZnO each contained 43% O_L_. The large O_L_ fraction in P-ZnO confirmed its good crystallinity, consistent with intense feature B in the XAS spectrum and the high signal-to-noise ratio in the XRD pattern. CA-ZnO and SWV-ZnO exhibited similar O_V_ fractions that were higher than that of P-ZnO, matching the EPR results, which reveals that the two electrochemical reconstruction approaches generated oxygen vacancies in the near-surface Zn–O–Zn layer.

### 3.2 CO_2_RR Performance

To further understand the electrocatalytic performance of the samples after electrochemical surface reconstruction, panels a–c of Figure 4 illustrate the LSV curves of P-ZnO, CA-ZnO, and SWV-ZnO, respectively, under N_2_ and CO_2_ atmospheres, allowing direct assessment of their potential toward CO_2_RR performance. At higher overpotentials (less than −1.05 V vs RHE), CA-ZnO and SWV-ZnO showed notably enhanced current densities in CO_2_ compared to N_2_. This observation suggested that both CA-ZnO and SWV-ZnO favored the CO_2_RR over the HER. Moreover, SWV-ZnO clearly showed a distinct reduction peak under the N_2_ atmosphere. This reduction peak was presumably associated with the highly active Zn/ZnO heterointerfaces created by pulsed-potential reconstruction, facilitating easier self-reduction and structural reconstruction. However, under a CO_2_ atmosphere, the reduction peak of SWV-ZnO diminished significantly. This indicated that the presence of CO_2_ stabilized the SWV-ZnO surface, effectively suppressing extensive self-reduction and promoting the CO_2_RR instead. Such behavior implied an improved long-term stability of SWV-ZnO for the CO_2_RR and further suggested that abundant CO_2_ adsorption on the catalyst surface enhanced its selectivity toward the CO_2_RR.

**Figure 4:**
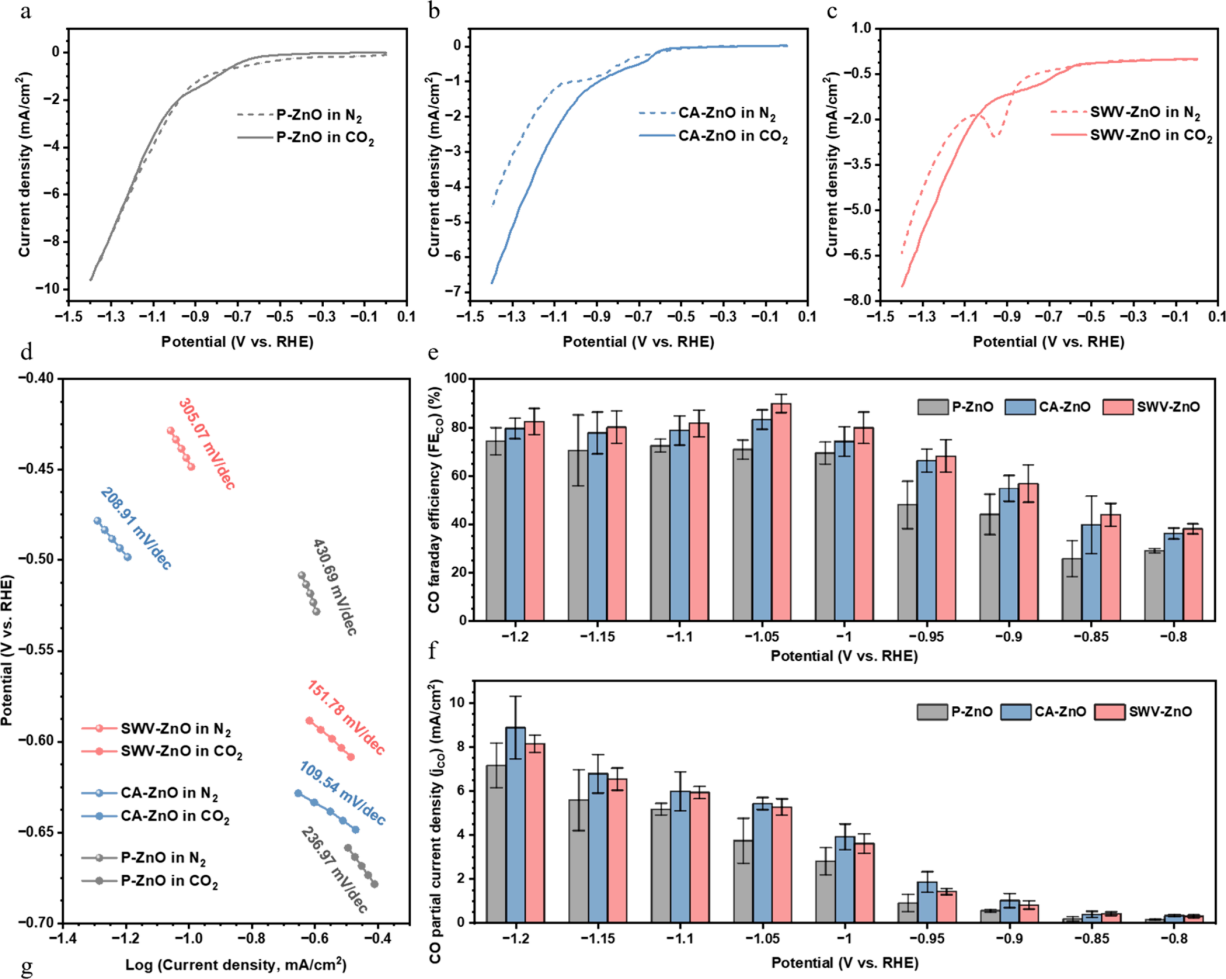
Linear-sweep voltammograms of (a) P-ZnO, (b) CA-ZnO, and (c) SWV-ZnO recorded in the N_2_- and CO_2_-saturated H-cell. (d) Tafel plots near the onset potential recorded in the N_2_- and CO_2_-saturated H-cell. (e) Faradaic efficiency and (f) partial current densities for CO recorded in the H-cell.

Tafel analyses were used to investigate the low-overpotential thermodynamic behaviors (Figure 4d and Figure S15a–c). The onset potentials for P-ZnO, CA-ZnO, and SWV-ZnO under CO_2_ conditions shifted to more negative values compared to those under N_2_, indicative of a typical HER poisoning effect.^37^ The Tafel slopes of P-ZnO, CA-ZnO, and SWV-ZnO under CO_2_ conditions were notably lower than those under N_2_ conditions, demonstrating that all samples exhibited improved electrochemical kinetics toward the CO_2_RR over the HER. P-ZnO displayed a more negative onset potential and a larger Tafel slope than CA-ZnO and SWV-ZnO, reflecting its inherently greater thermodynamic and kinetic barriers prior to both the HER and the CO_2_RR.

Panels e and f of Figure 4 display the Faradaic efficiencies for CO (FE_CO_) and partial current densities for CO (j_CO_) of P-ZnO, CA-ZnO, and SWV-ZnO at various applied potentials. The corresponding Faradaic efficiencies for H_2_ (FE_H_2__), total current densities (*j*_total_), and hydrogen partial current densities (*j*_H_2__) are provided in panels d–f, respectively, of Figure S15. As anticipated from the LSV and Tafel analyses, both CA-ZnO and SWV-ZnO exhibited higher FE_CO_ values than P-ZnO within the potential range of −0.8 to −1.2 V versus RHE (Figure 4e). At −1.05 V versus RHE, CA-ZnO and SWV-ZnO reached their peak FE_CO_ values of approximately 81% and 90%, respectively. Additionally, SWV-ZnO consistently exhibited FE_CO_ values higher than those of CA-ZnO across the entire potential range, underscoring the effectiveness of the pulsed-potential reconstruction approach in enhancing the CO_2_RR performance. Figure 4f shows that *j*_CO_ values for CA-ZnO and SWV-ZnO were comparable, and both significantly outperformed P-ZnO. It is also worth noting that the current of SWV-ZnO was lower than that of CA-ZnO.

### 3.3 Interfacial pH Evaluation during the CO_2_RR Process

After referencing previous studies on improving the CO_2_RR activity through electrochemical reconstruction, the improved CO_2_RR activity is generally attributed to the suppressed HER due to the increased specific surface area. The larger surface area produces a more pronounced increase in the local pH near the cathode, thus hindering the HER.^24^ Note that HER rates in acidic and alkaline media can differ by up to 2 orders of magnitude. For further explanation, the overall CO_2_RR pathway involves two proton-coupled electron transfer (PCET) steps, which proceed according to eqs 12–15 (an asterisk denotes an active site on the catalyst surface, and HA represents a proton donor):^38^

12 $$*+{\mathrm{CO}}_{2}\to {\mathrm{*CO}}_{2}$$

13 $${\mathrm{*CO}}_{2}+e^{-}+\mathrm{HA}\to \mathrm{*COOH}+A^{-}$$

14 $$\mathrm{*COOH}+e^{-}+\mathrm{HA}\to \mathrm{*CO}+H_{2}O+A^{-}$$

15 *CO→*+CO

Under typical conditions, water rather than the solvated H^+^ ion serves as the primary proton donor, which explains why the surface pH has a modest influence on the intrinsic kinetics of CO_2_ reduction. A previous study by Gupta et al. showed that changes in the electrochemical surface area (ECSA) regulate the HER rate through interfacial pH over the CO_2_RR pathway.^24^ To decouple the effect of surface area, CV measurements were conducted at 200 mV/s within the range of 0.3–0.4 V versus Ag/AgCl to obtain the ECSA factors, which are shown in Figure S16a. *j*_total_, *j*_CO_, and *j*_H_2__ for CA-ZnO and SWV-ZnO were divided by their respective ECSA coefficients, and the normalized values are given in Figure S16b and panels a and b of Figure 5. Figure 5a shows that the corrected *j*_CO_ (*j*_CO,ECSA_) of SWV-ZnO was almost identical to that of P-ZnO, indicating that they had similar intrinsic CO_2_ absorption activity. In contrast, CA-ZnO exhibited a lower *j*_CO,ECSA_. On the other hand, Figure 5b reveals *j*_H_2_,ECSA_ values for CA-ZnO and SWV-ZnO that were lower than the value for P-ZnO, which implied that the HER activity governed the FE_CO_ between the samples.

**Figure 5:**
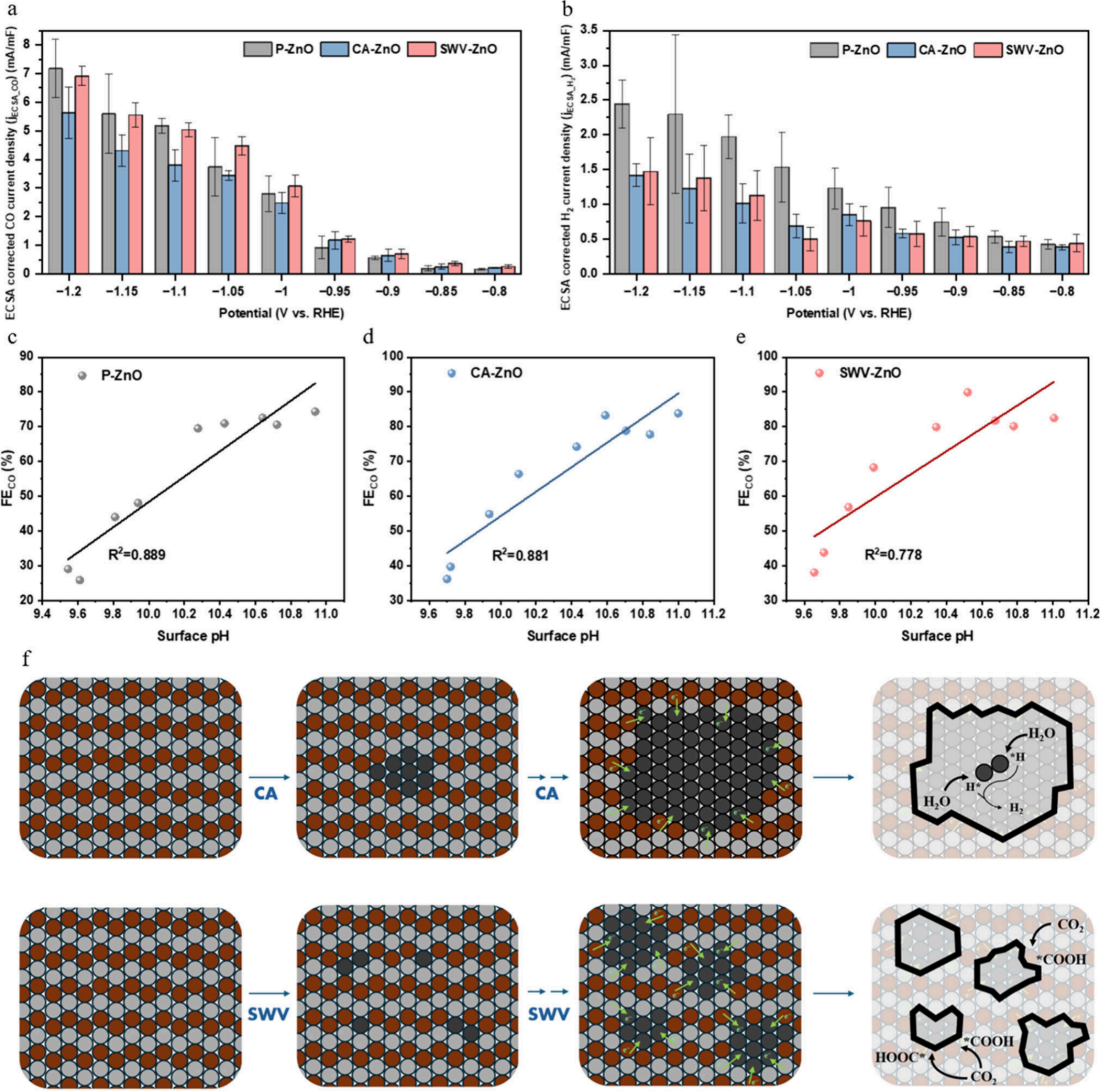
ECSA-corrected (a) CO and (b) H_2_ partial current density for P-ZnO, CA-ZnO, and SWV-ZnO. Correlation between simulated interfacial pH and FE_CO_ for (c) SWV-ZnO, (d) CA-ZnO, and (e) SWV-ZnO. (f) Schematic showing the growth mechanism of reconstructed metallic nano Zn clusters for CA-treated and SWV-treated samples.

To further decouple the HER activity from interfacial pH, we constructed a diffusion–reaction model that used the experimentally measured *j*_total_ to calculate the local pH at the cathode–electrolyte interface during electrolysis according to Gupta et al.,^23^ and the results are shown in Figure S17a–c. Panels c–e of Figure 5 plot the interfacial pH against FE_CO_ for P-ZnO, CA-ZnO, and SWV-ZnO, respectively, over a range of current densities, together with the coefficients of determination (*R* ^2^) obtained from linear fits. For P-ZnO and CA-ZnO, the highly linear relationship indicated that differences in FE_CO_ arose mainly from HER suppression associated with ECSA effects. However, SWV-ZnO showed a relatively weaker correlation. As a result, the higher FE_CO_ of CA-ZnO over P-ZnO was attributed to its larger ECSA, which increased the localized current density, as well as OH^–^ accumulation. This was consistent with the results reported by previous studies.^24^ Combined with the ECSA-corrected results, interfacial pH modeling and the XPS examinations together implied that the higher CO selectivity of SWV-ZnO could be attributed to the enriched Zn/ZnO heterointerfaces, which were relatively independent of the surface area and oxygen vacancies (Figure 5f). At the Zn/ZnO heterointerface, the charge transfer effect and the strain effect were the two most plausible mechanisms that could boost CO_2_RR performance.^32^ In the charge transfer scenario, electrons are extracted from ZnO into metallic Zn by the built-in interfacial field via the cathodic CO_2_RR.^33^ This electron redistribution enriched the electron density of interfacial Zn sites and depleted the density of neighboring ZnO sites. The extra charge localized on Zn sites would decrease the free energy of *COOH and simultaneously weaken *H adsorption, thereby accelerating CO desorption while suppressing the HER.^34^ This suggested that pulse-potential electrochemical reconstruction could better regulate heterointerface engineering in order to promote CO_2_RR activity. By introducing different pulse waveforms and their internal parameters, it was expected that better low-cost catalysts could be obtained.

Long-term stability is another important criterion for the CO_2_RR. The long-term performance was carried out in a gas-fed flow cell designed to emulate industrial operating conditions and to test the durability of the SWV-ZnO cathode. The highly alkaline anolyte (1 M KOH) used provided an abundant supply of OH^–^ ions that traversed the ion exchange membrane through the Grotthuss mechanism, which decreased cell resistance and restrained energy dissipation across the cell.^39^ Moreover, employing KOH instead of KHCO_3_ markedly suppressed CO_2_ crossover and therefore improved overall single-carbon utilization efficiency.^40^ During an 8 h galvanostatic session at −3.5 V, FE_CO_ remained above 80% with limited fluctuations (Figure 6a), though a slight decrease was observed after 8 h. To verify whether SWV-ZnO preserved its Zn/ZnO heterointerfaces after long-term operation, grazing incidence XRD (GIXRD) was performed on the electrodes before and after stability testing (Figure S17), with the 2θ range of 30–60° magnified in panels b and c of Figure 6. Clearly, even after 8 h, both ZnO and Zn reflections were observed for SWV-ZnO (Figure 6c), confirming the persistence of heterointerfaces. However, the pronounced carbon substrate signal and attenuated ZnO peaks in Figure S18 suggest partial electrolyte-induced erosion or peeling of the catalyst layer during the CO_2_RR, which may account for the small decrease in FE_CO_. The HRTEM image further revealed that Zn(101) domains expanded after electrolysis for 8 h and, in extreme cases, evolved into large Zn(101) patches (Figure S19), evidencing surface reconstruction. Nevertheless, the highly alkaline electrolyte environment and the continuous OH^–^ accumulation during CO_2_RR enabled SWV-ZnO to sustain stable FE_CO_ throughout the 8 h operation.^24^

**Figure 6:**
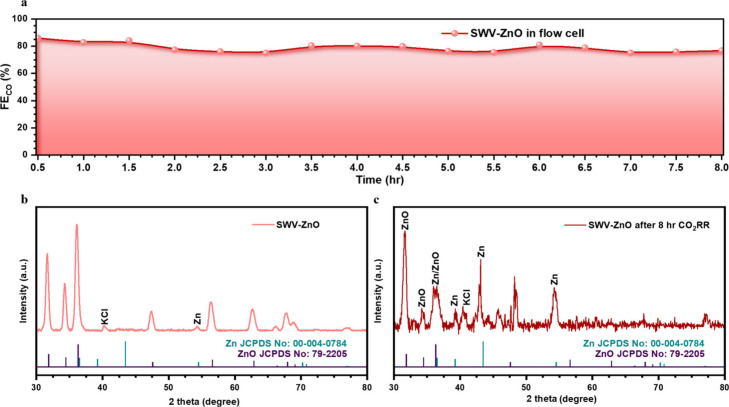
(a) Eight-hour durability test of SWV-ZnO recorded in a flow cell. GI XRD within the 2θ range of 30–60° magnified for SWV-ZnO (b) before and (c) after the CO_2_RR for 8 h.

## 4 Conclusion

In summary, we reported a simple yet effective pulsed-potential electrochemical reconstruction approach to engineer enhanced Zn/ZnO heterointerfaces that improve the CO_2_RR performance. The SWV reconstruction nucleated dispersed Zn nanoislands while preserving the underlying wurtzite ZnO lattice, thus improving the density of Zn/ZnO heterointerfaces and, through electron flow from ZnO to Zn at these heterointerfaces, likely stabilized *COOH while weakening *H. SWV-ZnO achieved a peak FE_CO_ of 90% at −1.05 V versus RHE in the H-cell. These CO_2_RR performances were better than those of the pristine zinc nanoparticles and those of the previously reported potentiostatic reconstruction (CA-ZnO). The flow cell test showed that SWV-ZnO maintained an FE_CO_ of ≈80 % for at least 8 h at an industrially relevant cell voltage of −3.5 V.

## Supplementary Material



## Notes

Schematic of the SWV protocol; SEM images of CA-ZnO and SWV-ZnO; Zn K-edge EXAFS, FIB cross sections, and TEM, SAED, and HRTEM data for all samples; Zn/ZnO heterointerface identification; Zn(101) mapping and TEM-based size quantification for CA-ZnO and SWV-ZnO; XPS survey (Zn 2p3, O 1s), H-cell electrochemistry (LSV, FE_H_2__, *j*_total_, *j*_H_2__, and ECSA-normalized *j*), and computed surface pH for all samples; GI-XRD data of SWV-ZnO before and after the CO_2_RR for 8 h; post-CO_2_RR HRTEM/FFT data; and TEM size quantification method (PDF)
