# Supplementary material for: Enhanced Zn/ZnO Heterointerfaces via Pulsed-Potential Electrochemical Reconstruction for Highly Selective CO2 Reduction
Source: ACS Appl Mater Interfaces. 2025 Oct 2;17(41):56991–7001. doi: 10.1021/acsami.5c11775 (PMC12532096; doi:10.1021/acsami.5c11775)
Supplement: Supplementary file 1 [file am5c11775_si_001.pdf]

Supporting Information

Enhanced Zn/ZnO Heterointerfaces via Pulsed-Potential Electrochemical Reconstruction for Highly Selective CO_2 Reduction

Hsin-Chiao Wu[†,1], Yu-Wei Huang[†,1], Yu-Chang Lin[‡], Chu-Hsin Yang[†], Ta-Chung Liu[†,*]

† Department of Biomedical Engineering, National Yang Ming Chiao Tung University, Taipei 112, Taiwan.

‡ National Synchrotron Radiation Research Center, Hsinchu 30076, Taiwan.

1 These first authors contributed equally: Hsin-Chiao Wu, Yu-Wei Huang

[*]Corresponding author's e-mail: tcliu@nycu.edu.tw

Figure S1. Schematic of the square-wave voltammetry protocol.

Figure S2. High-magnification SEM inspection of (a) CA- ZnO and (b) SWV-ZnO.

Figure S3. (a) k space Zn K-edge EXAFS and (b) Fourier-transformed Zn K-edge EXAFS spectra of P-ZnO, CA-ZnO and SWV-ZnO.

Figure S4. FIB-prepared cross-sections of (a) CA-ZnO and (b) SWV-ZnO on gas-diffusion electrodes. TEM images and corresponding selected area electron diffraction patterns for (c) CA-ZnO and (d) SWV-ZnO. (e) HR-TEM images and (f) corresponding SAED pattern for P-ZnO.

Figure S5. Identification of Zn/ZnO heterointerface for (a) (i)-(v) CA-ZnO and (b) (i)-(v) SWV-ZnO.

Figure S6. (a-i) (b-i) HR-TEM images, (a-ii) (b-ii) FFT images, and (a-iii) (b-iii) corresponding Zn(101) regions in HR-TEM and (a-iv) (b-iv) FFT images for CA-ZnO.

Figure S7. (a-i) (b-i) HR-TEM images, (a-ii) (b-ii) FFT images, and (a-iii) (b-iii) corresponding Zn(101) regions in HR-TEM and (a-iv) (b-iv) FFT images for SWV-ZnO.

Figure S8. (a)-(c) (i) CA-ZnO HRTEM image for TEM-based particle size quantification; (a)-(c) (ii) FFT images; (a)-(c) (iii) FFT images marked with orange for corresponding Zn(101) regions.

Figure S9. (a)-(c) (i) CA-ZnO HRTEM image for TEM-based particle size quantification; (a)-(c) (ii) FFT images; (a)-(c) (iii) FFT images marked with orange for corresponding Zn(101) regions.

Figure S10. (a)-(c) (i) CA-ZnO HRTEM image for TEM-based particle size quantification; (a)-(c) (ii) FFT images; (a)-(c) (iii) FFT images marked with orange for corresponding Zn(101) regions.

Figure S11. (a-i) SWV-ZnO HRTEM image for TEM-based particle size quantification; (a-ii) FFT images; (a-iii) FFT images marked with orange for corresponding Zn(101) regions.

Figure S12. Deconvoluted Zn $2p^3$ spectra of P-ZnO, fitted with Zn(0) (1020.9 eV), and Zn(II) (1021.9 eV).

Figure S13. Full XPS spectra for (a) P-ZnO, (b) CA-ZnO, and (c) SWV-ZnO.

Figure S14. Deconvoluted O 1s spectra of (a) P-ZnO, (b) CA-ZnO and (c) SWV-ZnO, fitted with lattice oxygen ($O_L \approx 530.2$ eV), vacancy-related oxygen ($O_V \approx 531.4$ eV) and adsorbed oxygen ($O_C \approx 532.6$ eV).

Figure S15. Low-over-potential linear-sweep voltammograms recorded in N_2 and CO_2 atmospheres for (a) P-ZnO, (b) CA-ZnO and (c) SWV-ZnO. (d) Faradaic efficiency for H_2, (e) total current density and (f) H_2 partial current density recorded in H-cell.

Figure S16. (a) Cyclic-voltammetry curves ofP-ZnO, CA-ZnO, and SWV-ZnO collected at 200 mV/s within 0.30–0.40 V vs. Ag/AgCl. (b) ECSA-normalised total current density for P-ZnO, CA-ZnO, and SWV-ZnO.

Figure S17. Calculated surface pH for (a) P-ZnO, (b) CA-ZnO and (c) SWV-ZnO.

Figure S18. GI XRD full spectra of SWV-ZnO (a) before and (a) after 8 hr CO_2RR.

Figure S19. (a-i) (b-i) HR-TEM images, (a-ii) (b-ii) FFT images, and (a-iii) (b-iii) corresponding Zn(101) regions in HR-TEM and (a-iv) (b-iv) FFT images for SWV-ZnO after 8 hr CO_2RR.

Figure S20. (a-i) HR-TEM images, (a-ii) FFT pattern, (a-iii) masked FFT pattern, (a-iv) IFFT image for masked FFT pattern, (a-v) measurement of lattice fringe, and (a-vi) corresponding Zn(101) regions in HR-TEM based on IFFT image for CA-ZnO.

Note S1. Method for TEM-based particle size quantification.

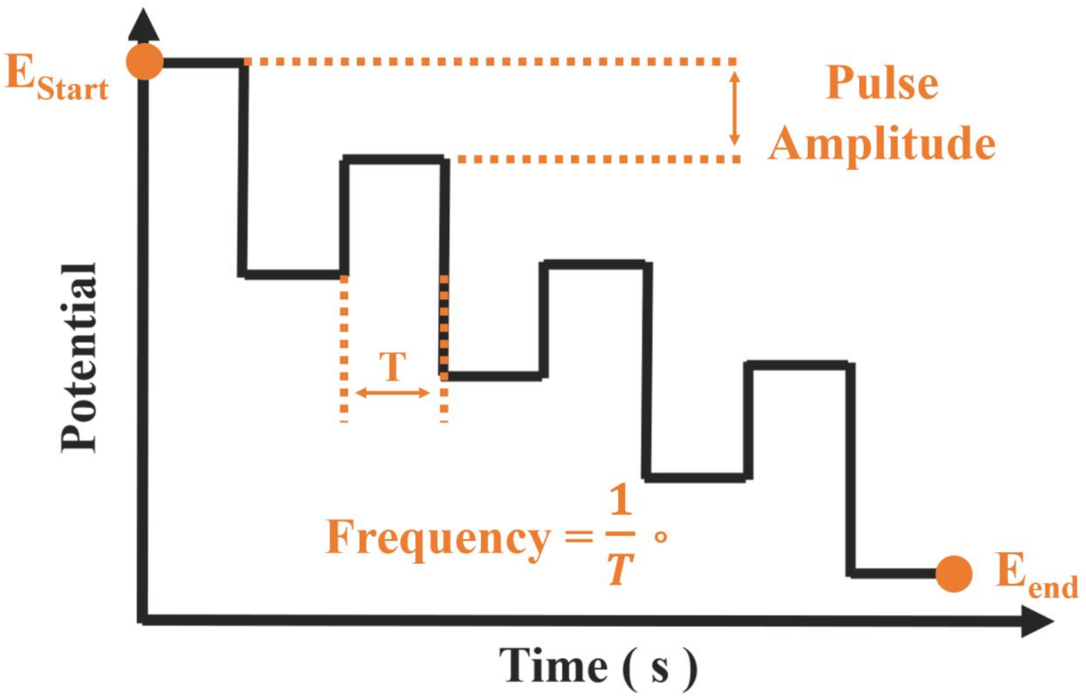

Figure S1. Schematic of the square-wave voltammetry protocol.

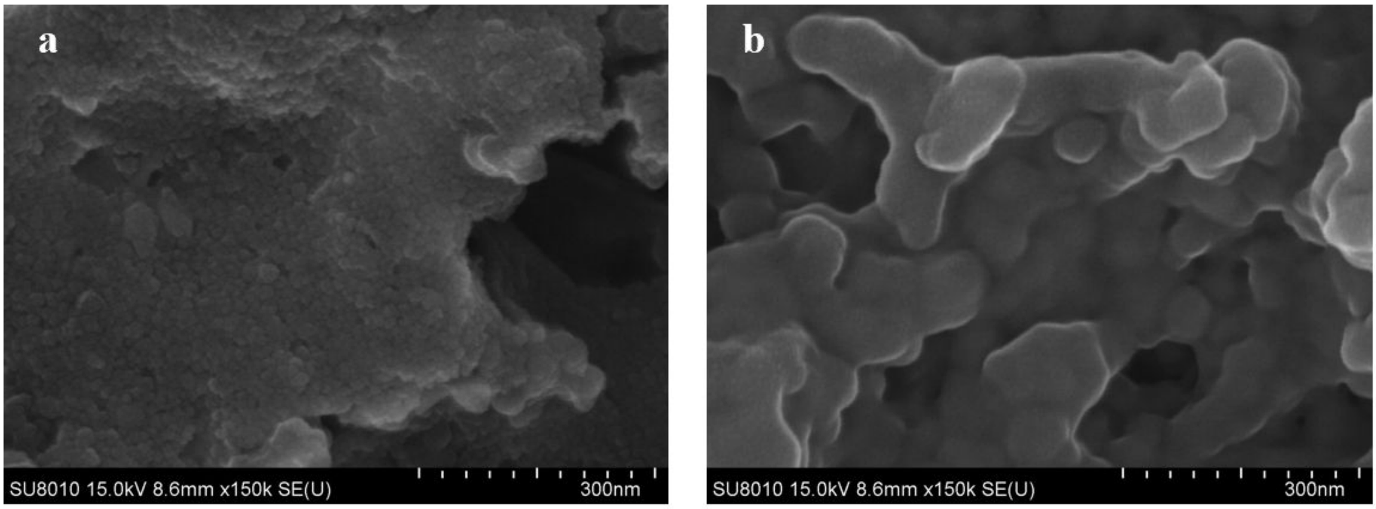

Figure S2. High-magnification SEM inspection of (a) CA- ZnO and (b) SWV-ZnO.

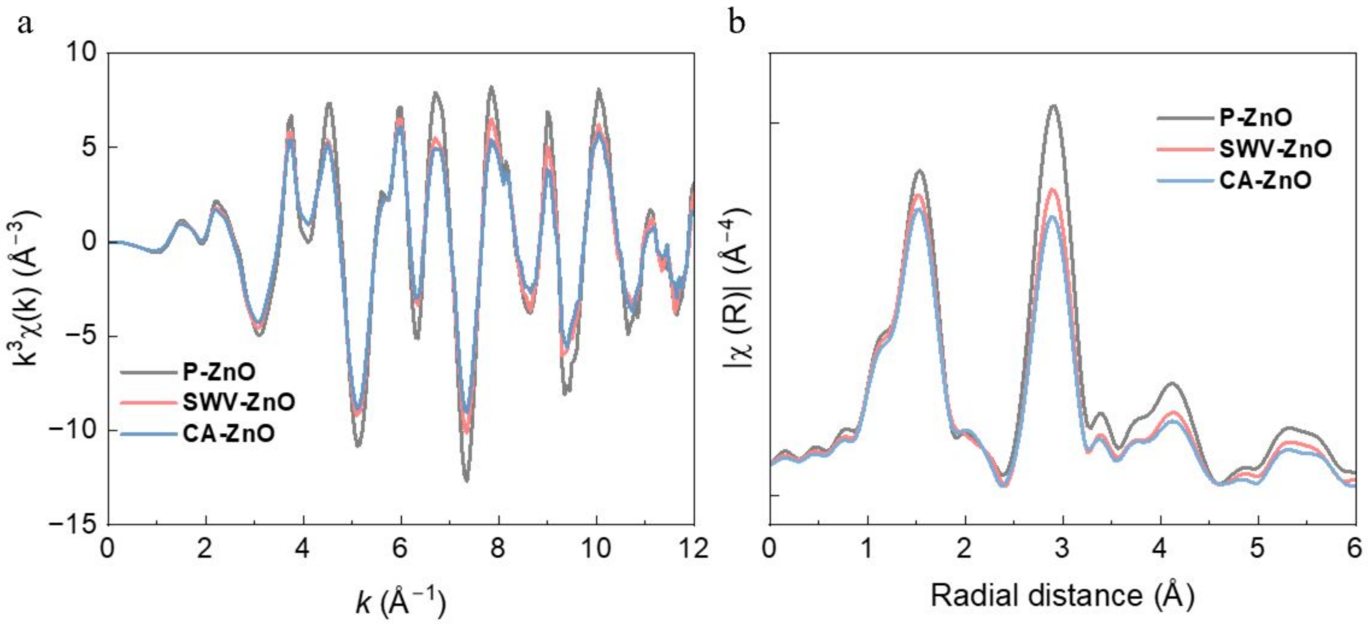

Figure S3. (a) k space Zn K-edge EXAFS and (b) Fourier-transformed Zn K-edge EXAFS spectra of P-ZnO, CA-ZnO and SWV-ZnO.

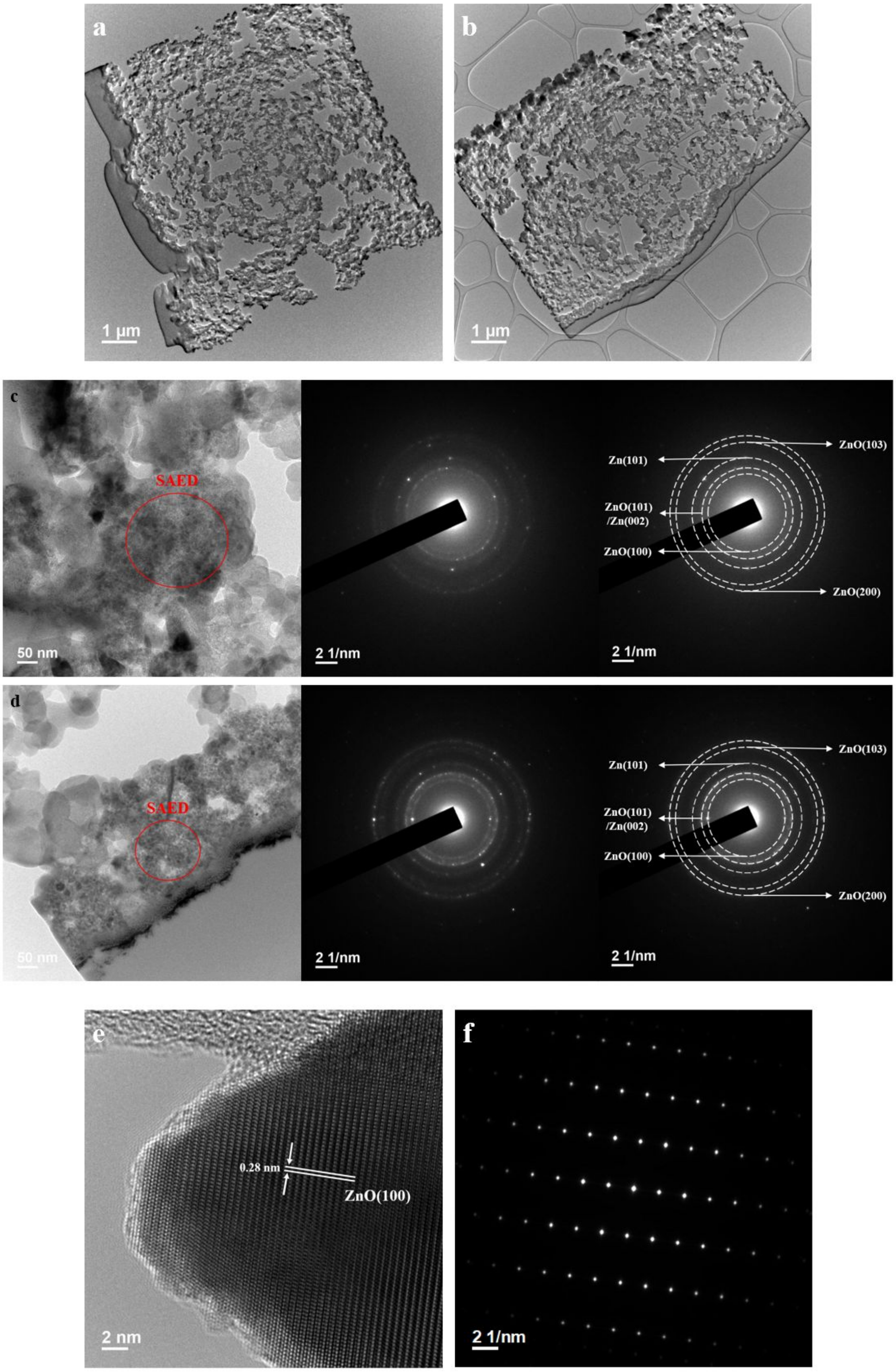

Figure S4. FIB-prepared cross-sections of (a) CA-ZnO and (b) SWV-ZnO on gas-diffusion electrodes. TEM images and corresponding selected area electron diffraction patterns for (c) CA-ZnO and (d) SWV-ZnO. (e) HR-TEM images and (f) corresponding SAED pattern for P-ZnO.

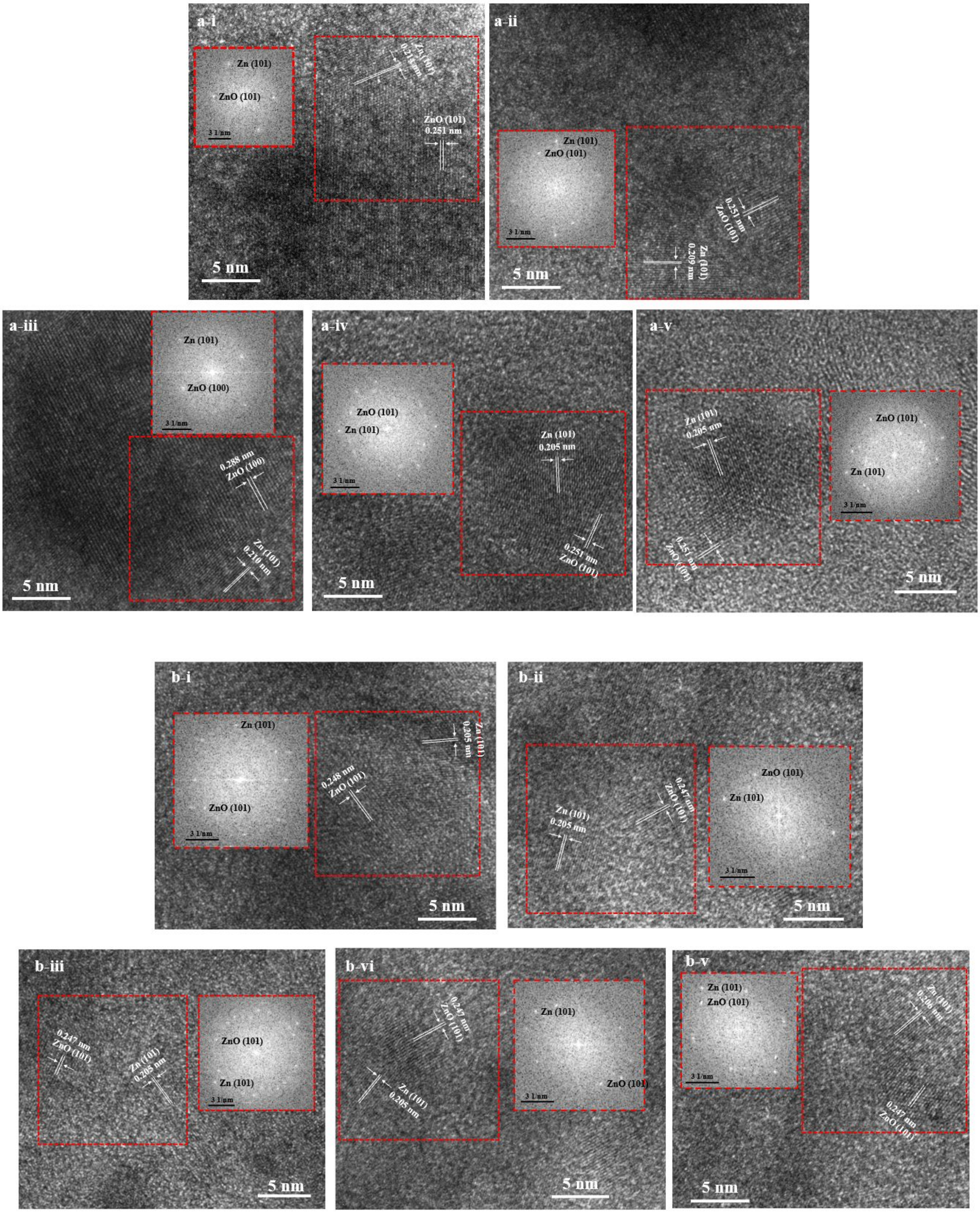

Figure S5. Identification of Zn/ZnO heterointerface for (a) (i)-(v) CA-ZnO and (b) (i)-(v) SWV-ZnO.

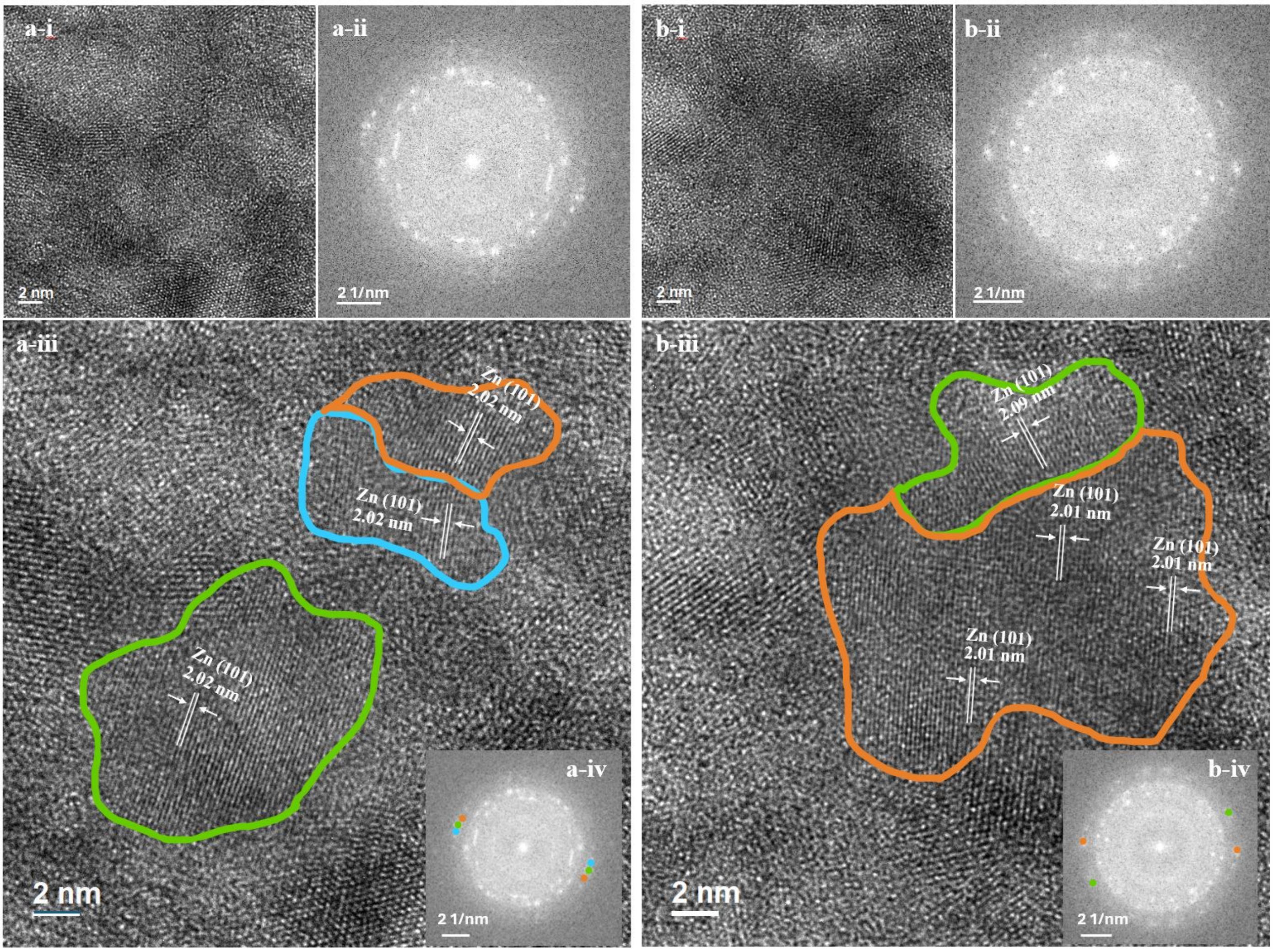

Figure S6. (a-i) (b-i) HR-TEM images, (a-ii) (b-ii) FFT images, and (a-iii) (b-iii) corresponding Zn(101) regions in HR-TEM and (a-iv) (b-iv) FFT images for CA-ZnO.

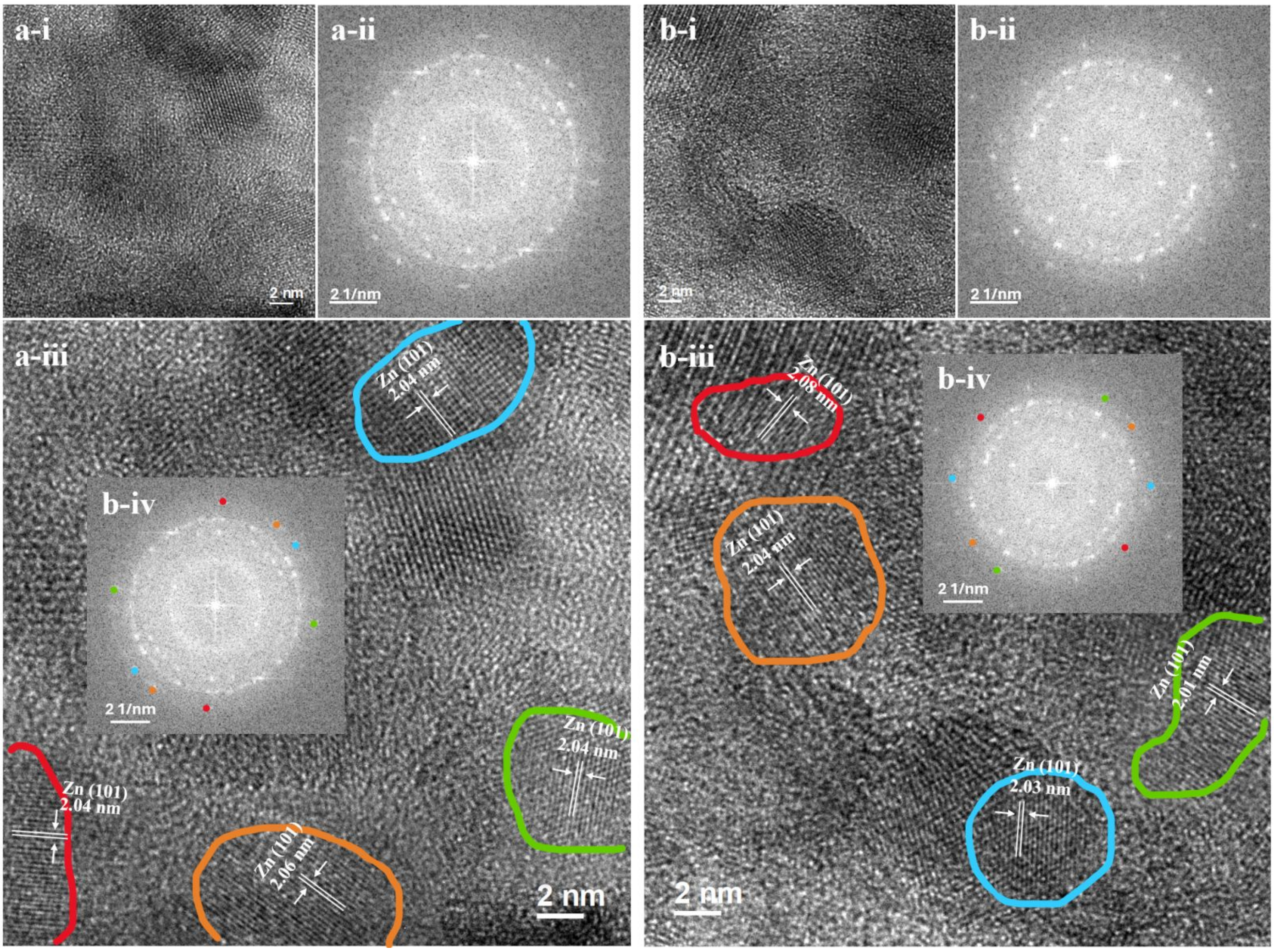

Figure S7. (a-i) (b-i) HR-TEM images, (a-ii) (b-ii) FFT images, and (a-iii) (b-iii) corresponding Zn(101) regions in HR-TEM and (a-iv) (b-iv) FFT images for SWV-ZnO.

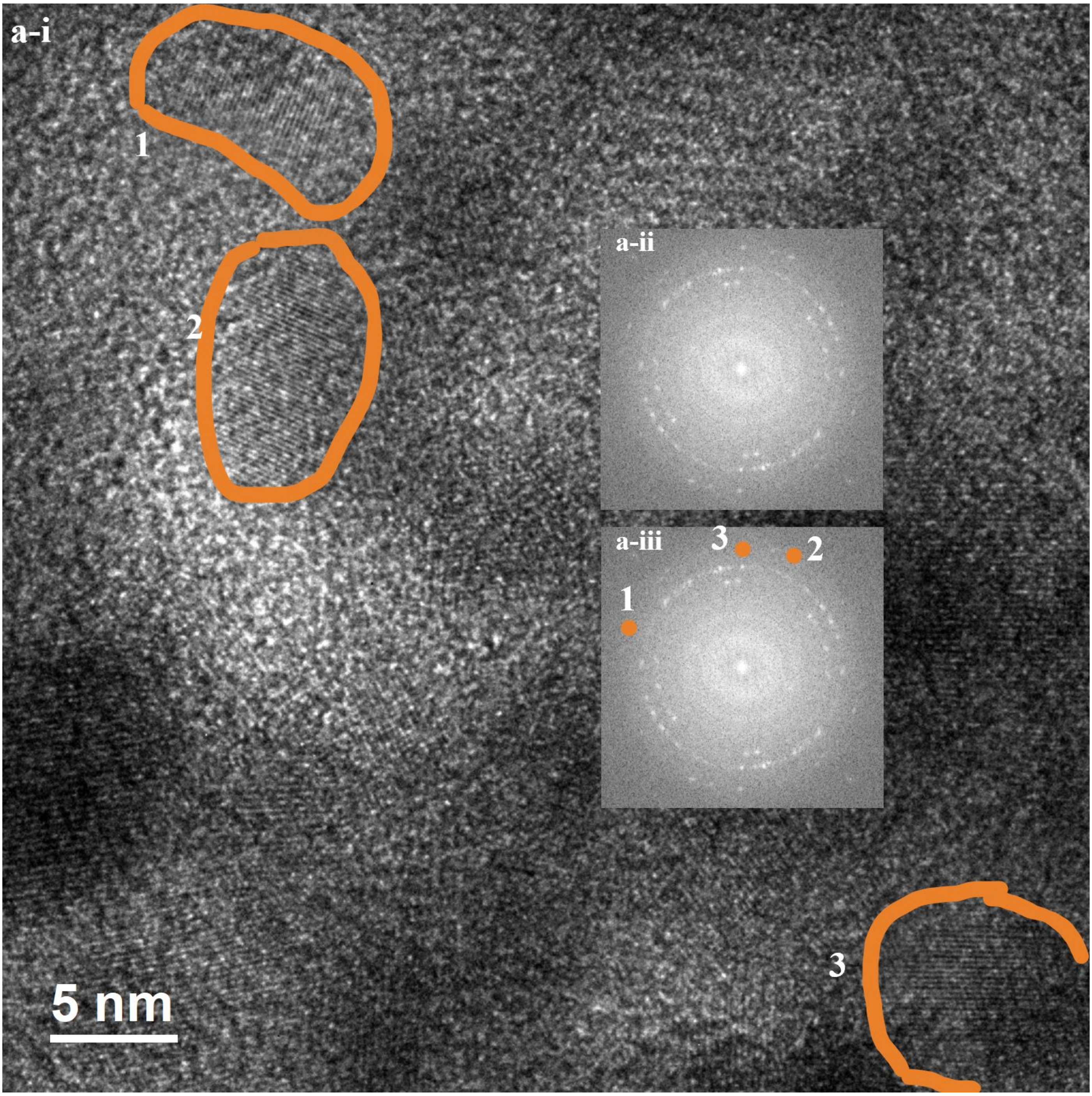

Figure S8. (a-i) CA-ZnO HRTEM image for TEM-based particle size quantification; (a-ii) FFT images; (a-iii) FFT images marked with orange for corresponding Zn(101) regions.

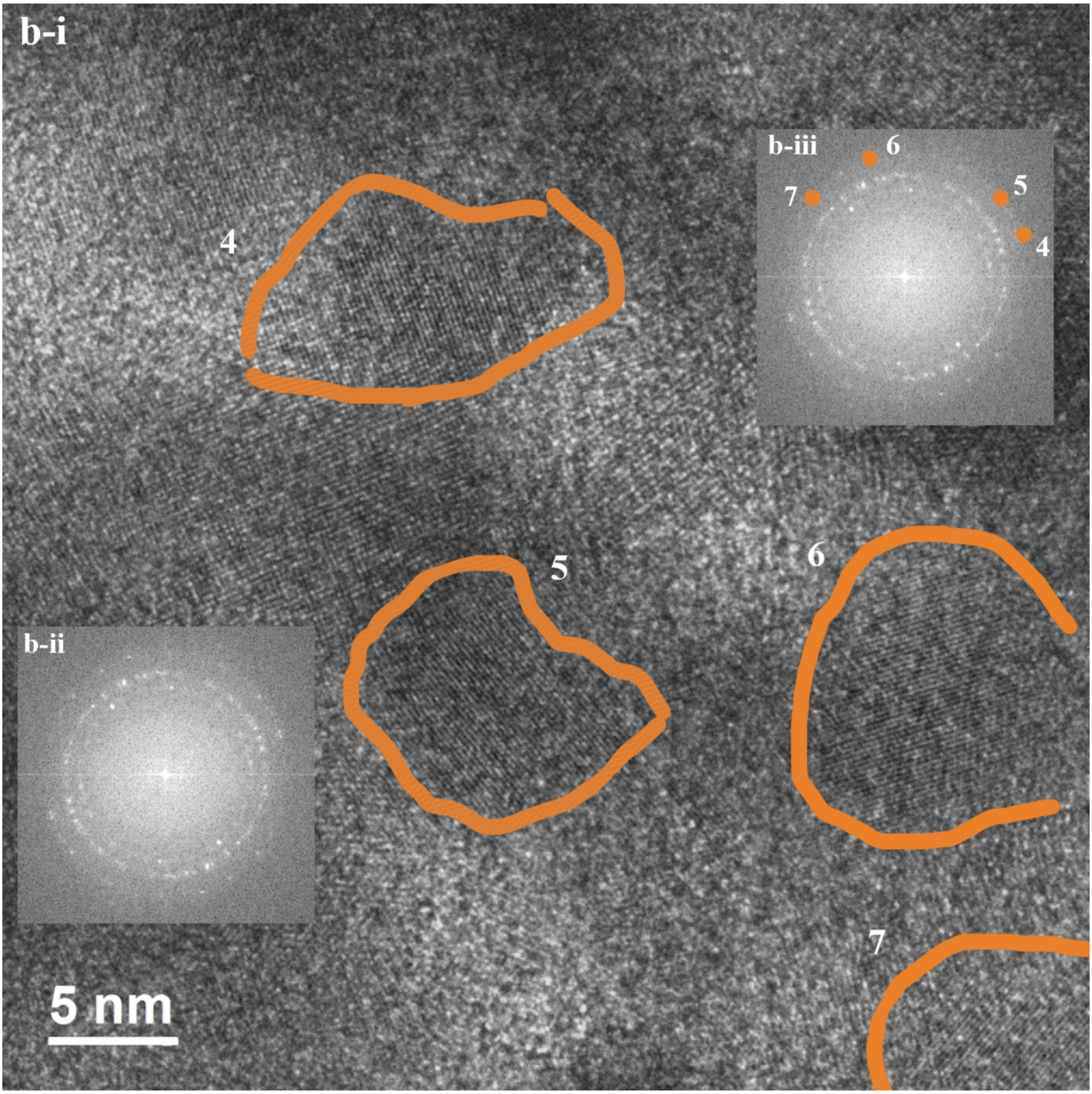

Figure S9. (b-i) CA-ZnO HRTEM image for TEM-based particle size quantification; (b-ii) FFT images; (b-iii) FFT images marked with orange for corresponding Zn(101) regions.

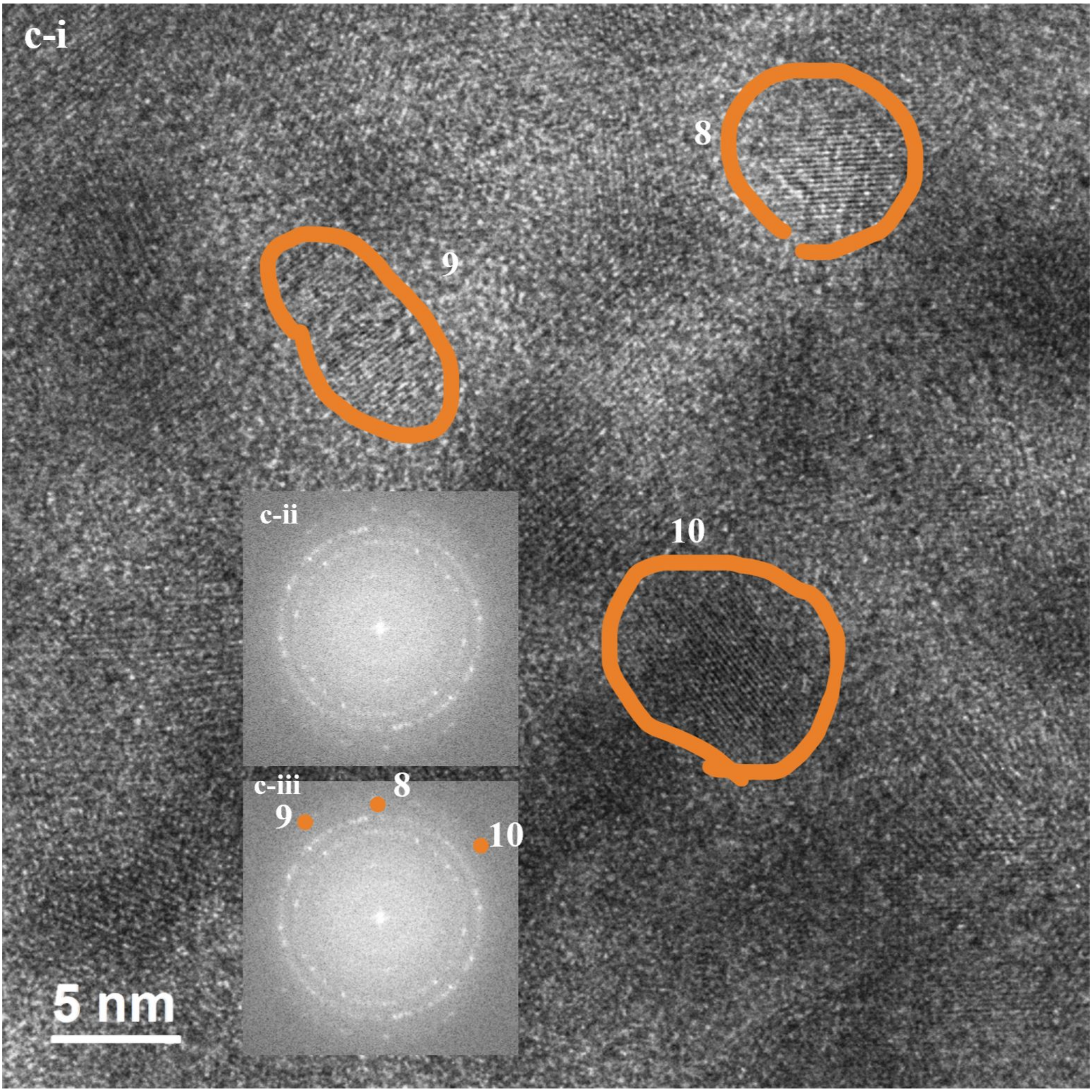

Figure S10. (c-i) CA-ZnO HRTEM image for TEM-based particle size quantification; (c-ii) FFT images; (c-iii) FFT images marked with orange for corresponding Zn(101) regions.

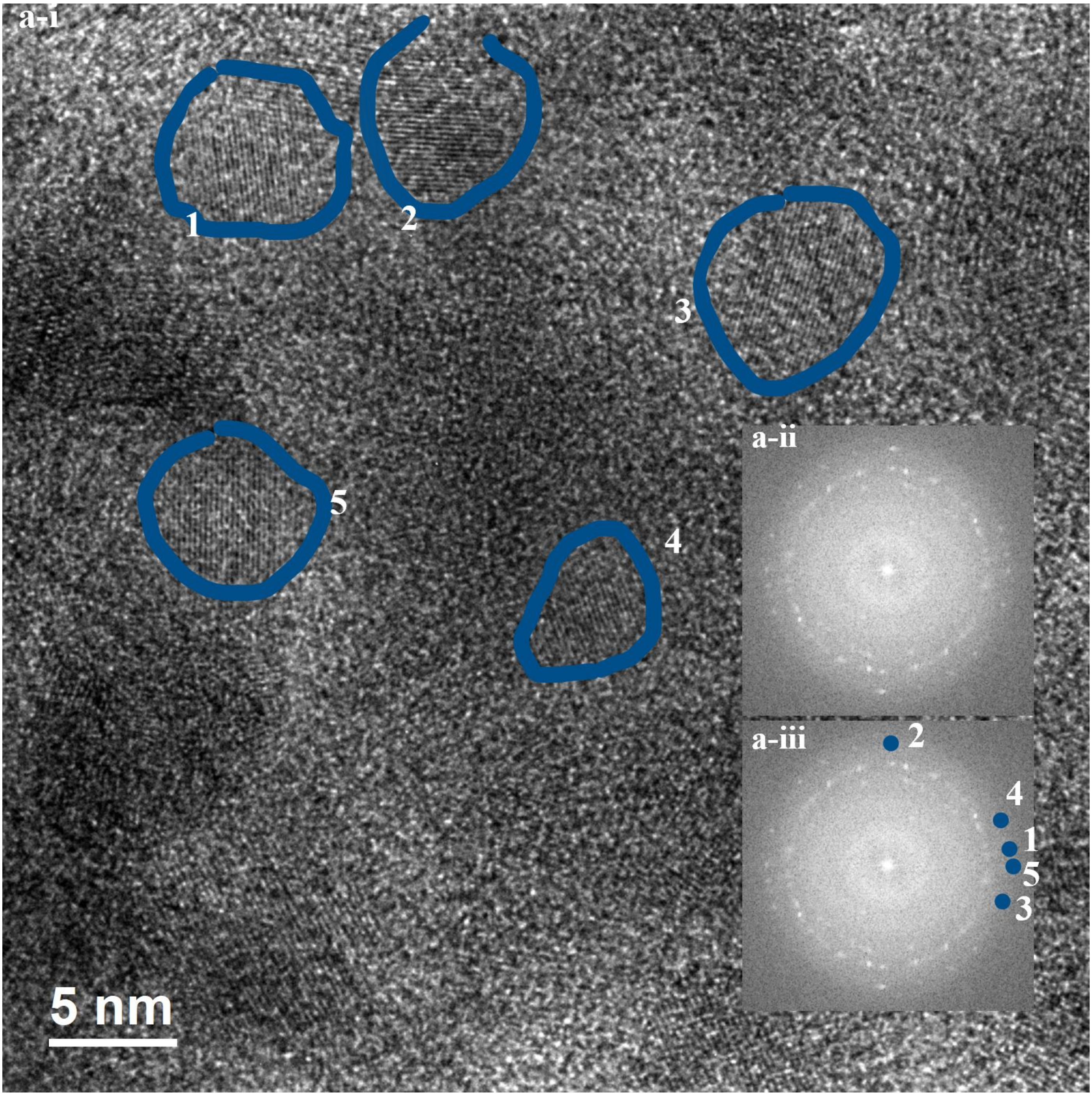

Figure S11. (a-i) SWV-ZnO HRTEM image for TEM-based particle size quantification; (a-ii) FFT images; (a-iii) FFT images marked with orange for corresponding Zn(101) regions.

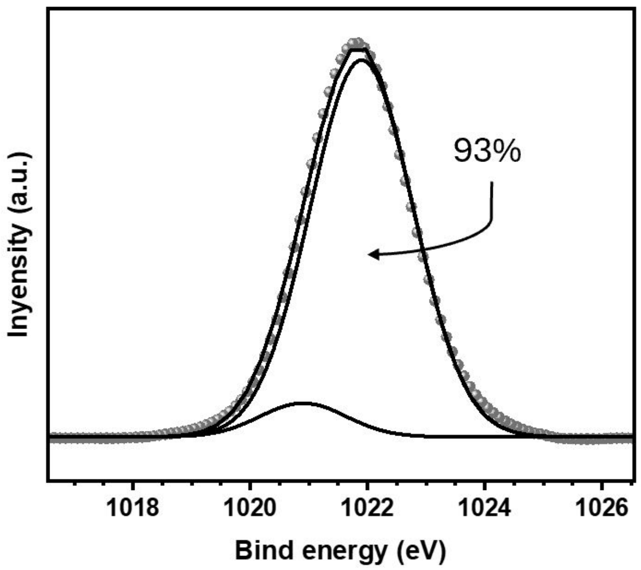

Figure S12. Deconvoluted Zn $2p^3$ spectra of P-ZnO, fitted with Zn(0) (1020.9 eV), and Zn(II) (1021.9 eV).

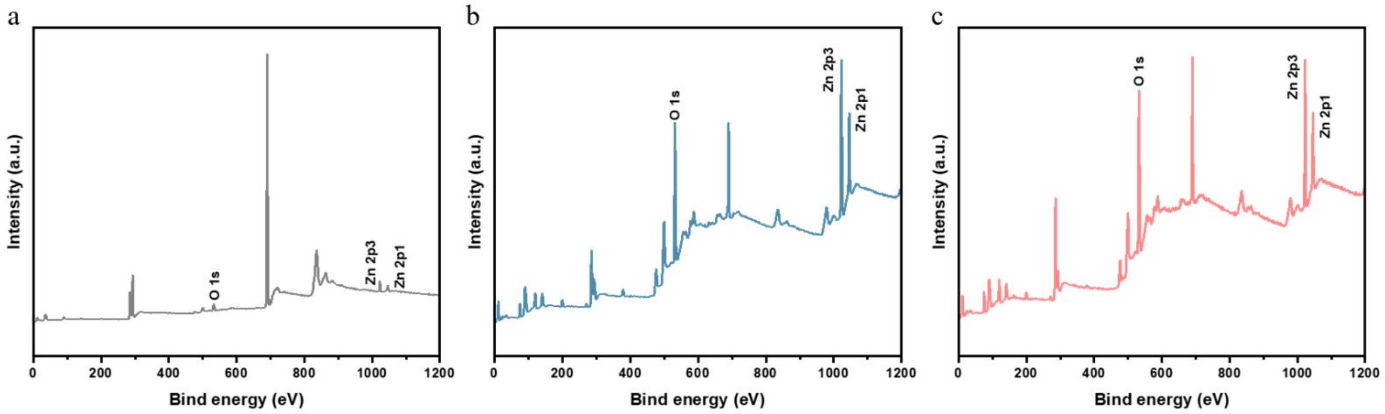

Fig S13. Full XPS spectra for (a) P-ZnO, (b) CA-ZnO, and (c) SWV-ZnO. All samples exhibited ZnO-characteristic peaks, namely the Zn $2p^3$ at 1022 eV, the Zn $2p^1$ at 1045 eV, and the O 1*s* at 531 eV.

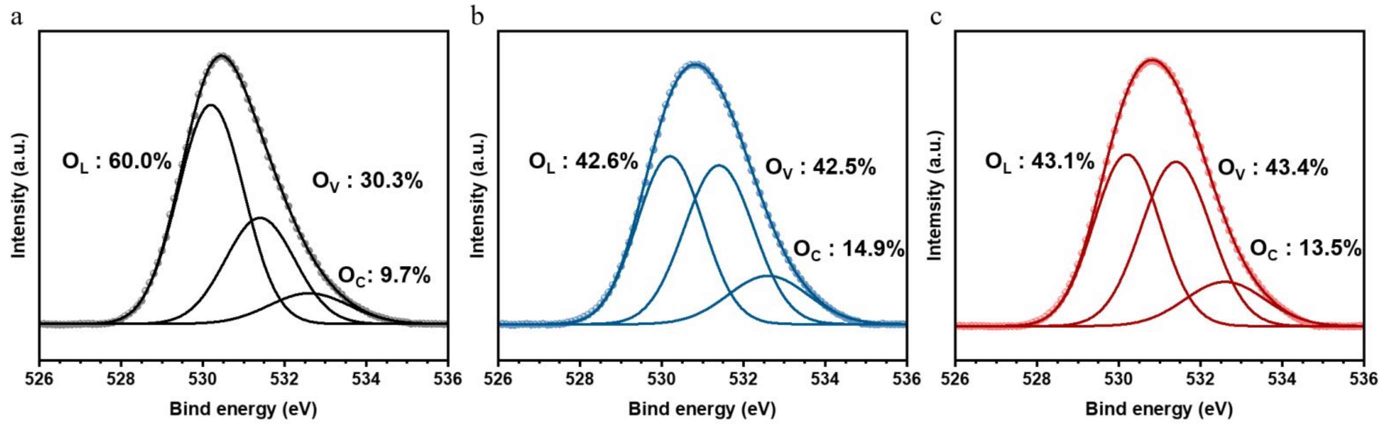

Fig S14. Deconvoluted O 1s spectra of (a) P-ZnO, (b) CA-ZnO and (c) SWV-ZnO, fitted with lattice oxygen ($O_L \approx 530.2$ eV), vacancy-related oxygen ($O_V \approx 531.4$ eV) and adsorbed oxygen ($O_C \approx 532.6$ eV).

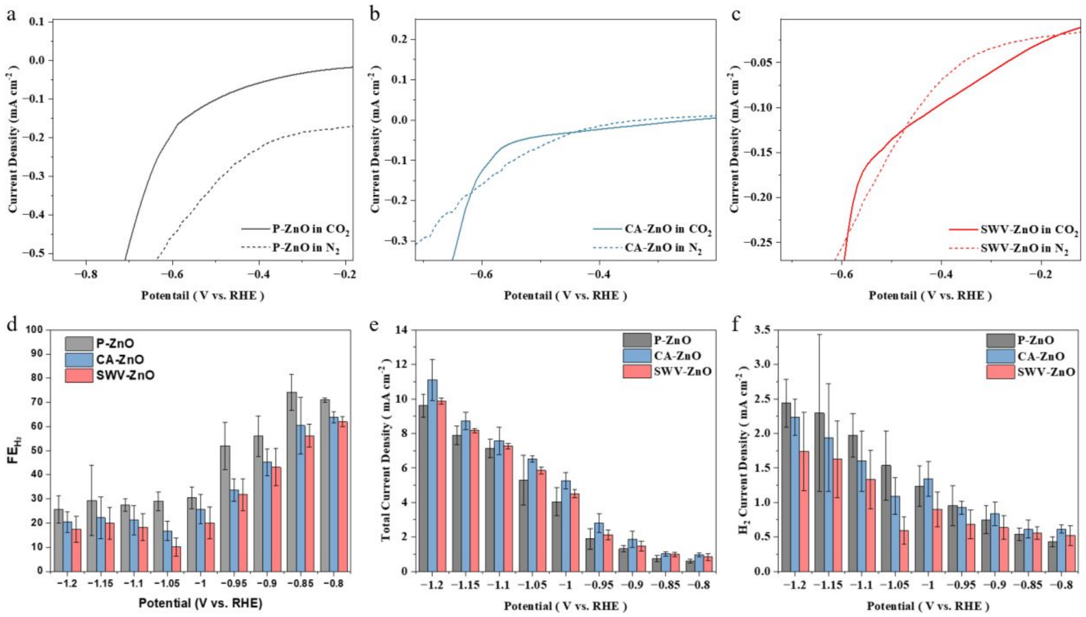

Figure S15. Low-over-potential linear-sweep voltammograms recorded in N_2 and CO_2 atmospheres for (a) P-ZnO, (b) CA-ZnO and (c) SWV-ZnO. (d) Faradaic efficiency for H_2, (e) total current density and (f) H_2 partial current density recorded in H-cell.

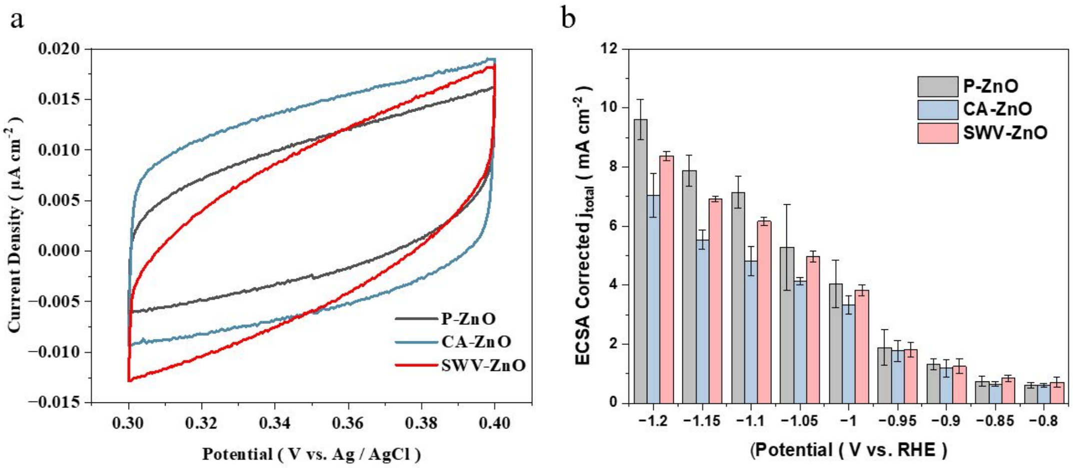

Figure S16. (a) Cyclic-voltammetry curves ofP-ZnO, CA-ZnO, and SWV-ZnO collected at 200 mV/s within 0.30–0.40 V vs. Ag/AgCl. (b) ECSA-normalised total current density for P-ZnO, CA-ZnO, and SWV-ZnO.

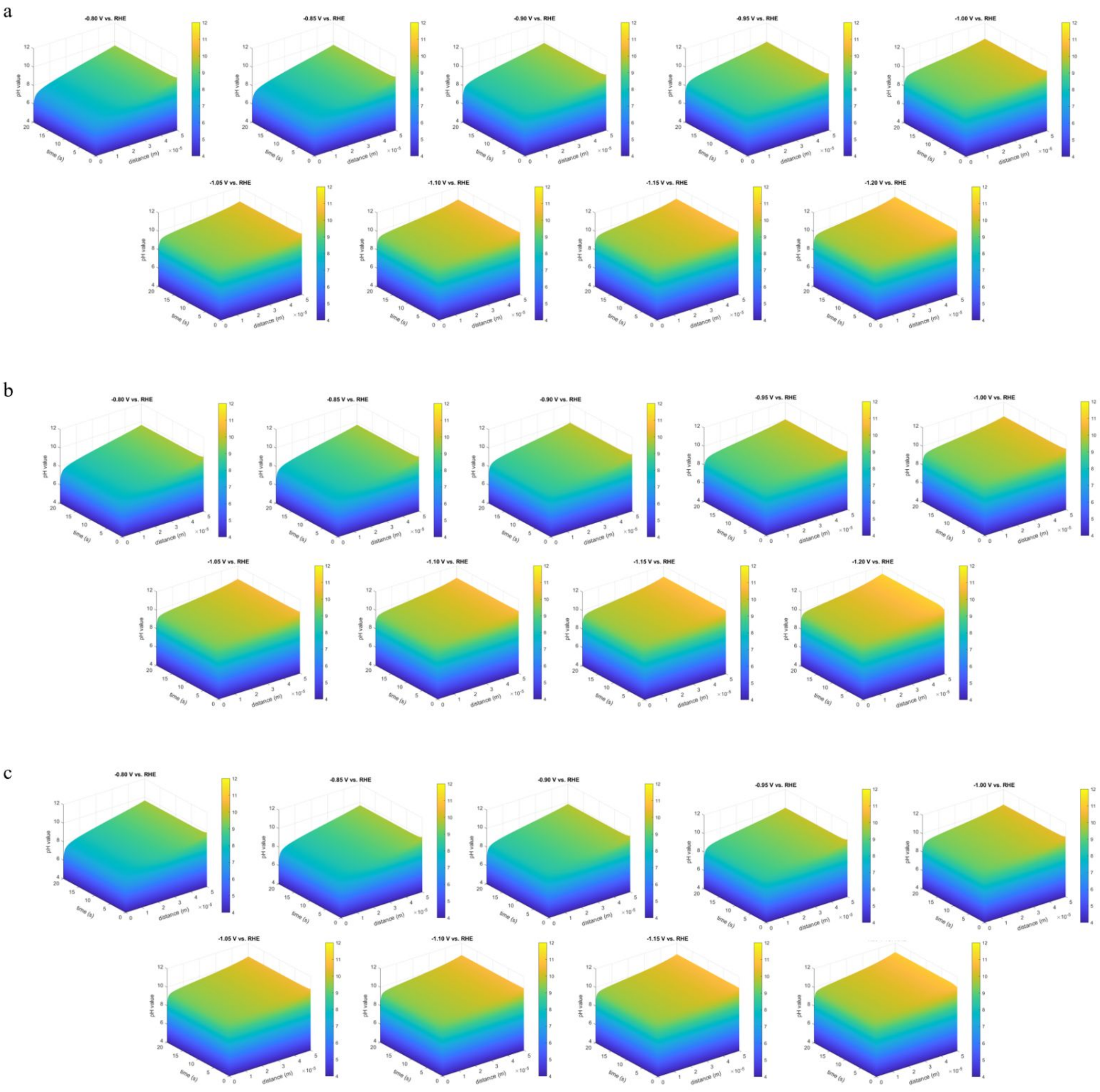

Figure S17. Calculated surface pH for (a) P-ZnO, (b) CA-ZnO and (c) SWV-ZnO.

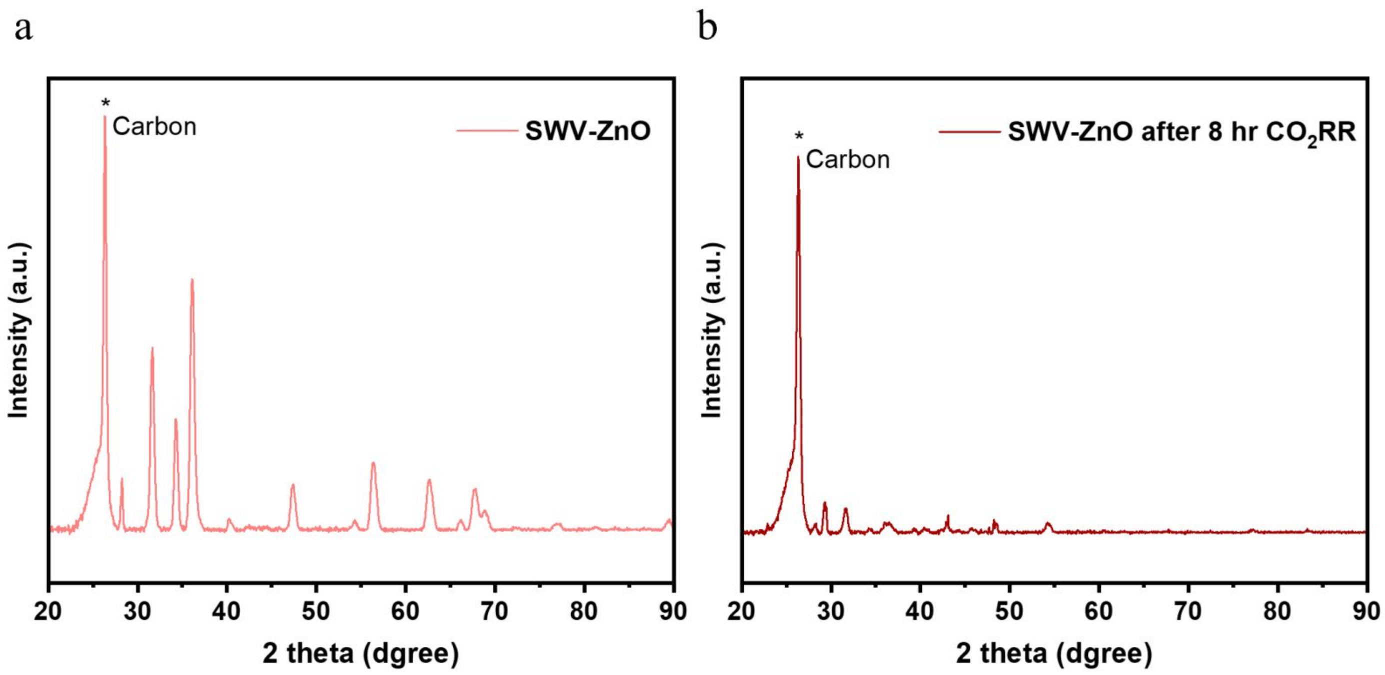

Figure S18. GI XRD full spectra of SWV-ZnO (a) before and (a) after 8 hr CO_2RR.

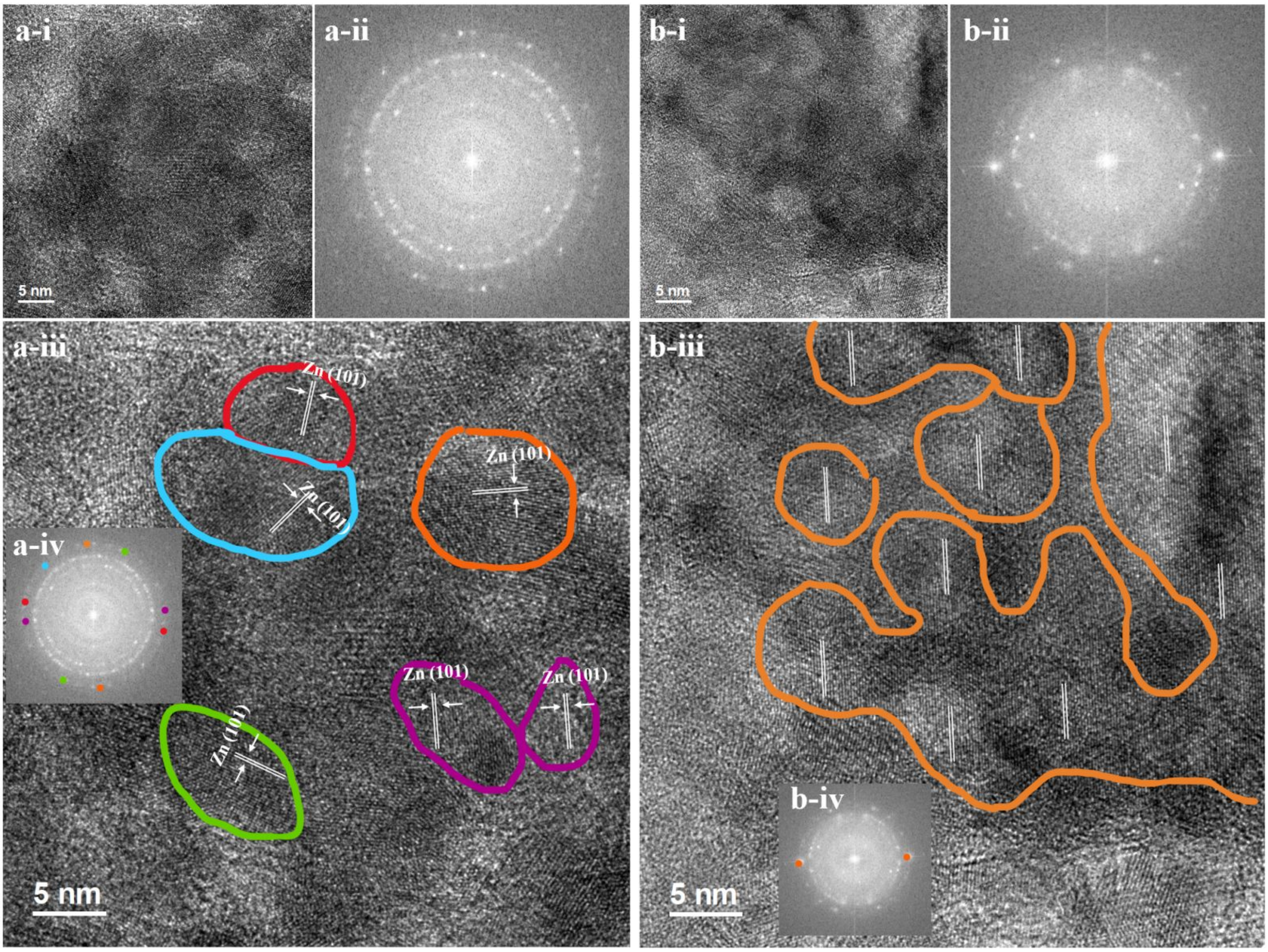

Figure S19. (a-i) (b-i) HR-TEM images, (a-ii) (b-ii) FFT images, and (a-iii) (b-iii) corresponding Zn(101) regions in HR-TEM and (a-iv) (b-iv) FFT images for SWV-ZnO after 8 hr CO_2RR.

Note S1. Method for TEM-based particle size quantification

The size of each Zn(101) domain was determined by masking the Zn(101) reflection in the FFT and then performing an inverse FFT (IFFT) in DigitalMicrograph software. For example, as illustrated in **Figure S20a**, panels **S20c-i** and **S20c-ii** present an HRTEM image of CA-ZnO and its corresponding FFT pattern, respectively. The prominent Zn(101) spot in **S20c-ii** was isolated with a mask (**S20c-iii**) and subjected to IFFT (**S20c-iv**). Regions exhibiting the strongest lattice-fringe intensity were then selected for measurement (**S20c-v**) and are marked in blue on the HRTEM image (**S20c-vi**).

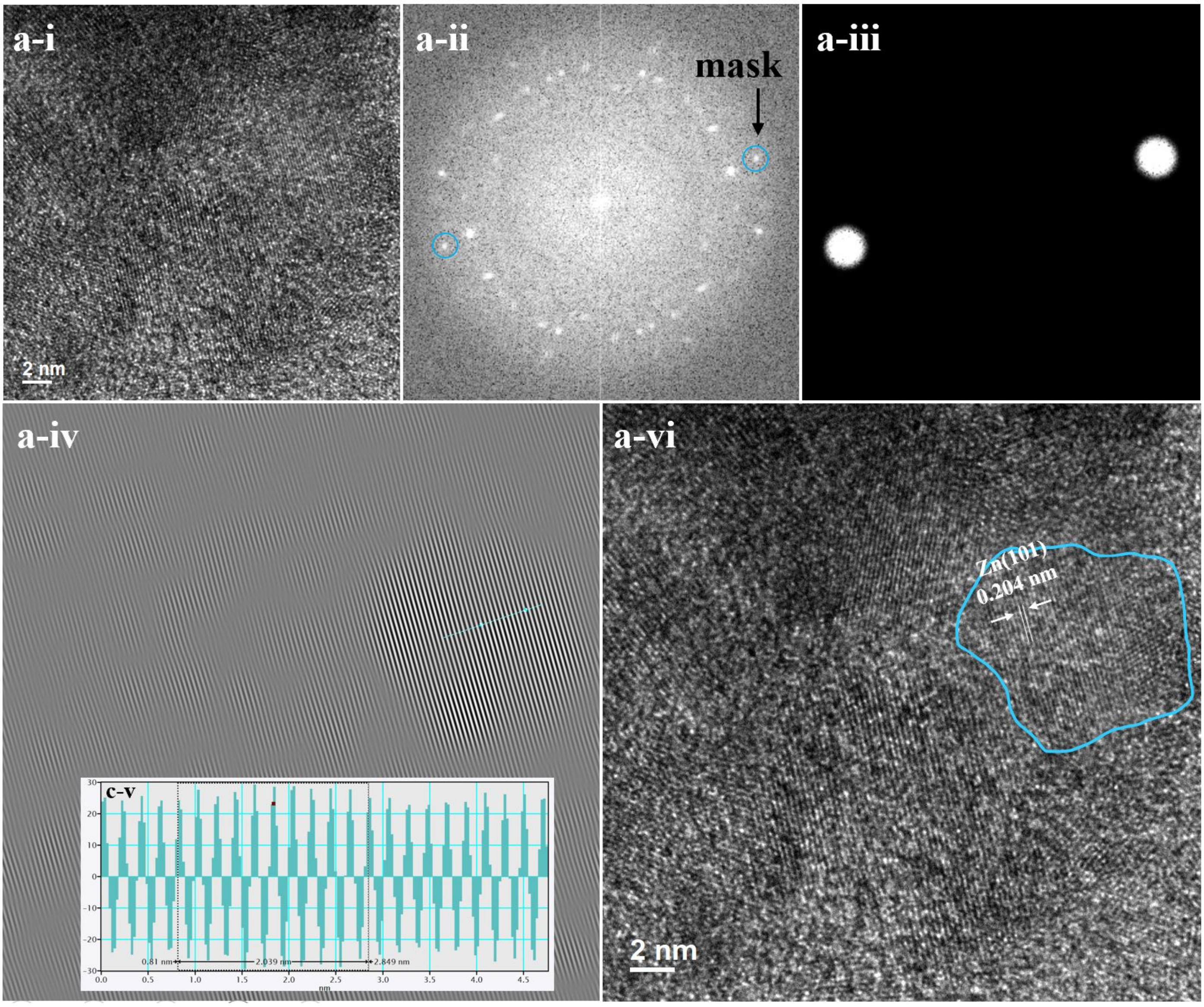

Figure S20. (a-i) HR-TEM images, (a-ii) FFT pattern, (a-iii) masked FFT pattern, (a-iv) IFFT image for masked FFT pattern, (a-v) measurement of lattice fringe, and (a-vi) corresponding Zn(101) regions in HR-TEM based on IFFT image for CA-ZnO.
